# Comparison of Cu(II) Adsorption Using Fly Ash and Natural Sorbents During Temperature Change and Thermal–Alkaline Treatment

**DOI:** 10.3390/ma18194552

**Published:** 2025-09-30

**Authors:** Anna Ďuricová, Veronika Štefanka Prepilková, Michal Sečkár, Marián Schwarz, Dagmar Samešová, Tomáš Murajda, Peter Andráš, Adriana Eštoková, Miriama Čambál Hološová, Juraj Poništ, Andrea Zacharová, Jarmila Schmidtová, Darina Veverková, Adrián Biroň

**Affiliations:** 1 Department of Environmental Engineering, Faculty of Ecology and Environmental Sciences, Technical University in Zvolen, T. G. Masaryka 24, 96001 Zvolen, Slovakia; xduricovaa@is.tuzvo.sk (A.Ď.); xseckarm@is.tuzvo.sk (M.S.); schwarz@is.tuzvo.sk (M.S.); samesova@is.tuzvo.sk (D.S.); tomas.murajda@mhth.sk (T.M.); zacharova@is.tuzvo.sk (A.Z.); 2 Fortischem a.s., M. R. Štefánika 1, 97271 Nováky, Slovakia; veronika.prepilkova@fortischem.sk; 3 Department of Geography and Geology, Matej Bel University in Banská Bystrica, Tajovského 40, 97401 Banská Bystrica, Slovakia; peter.andras@umb.sk; 4 Department of Material Engineering, Institute for Sustainable and Circular Construction, Faculty of Civil Engineering, Technical University of Košice, Vysokoškolská 4, 04200 Košice, Slovakia; adriana.estokova@tuke.sk (A.E.); miriama.cambal.holosova@tuke.sk (M.Č.H.); 5 Department of Mathematics and Descriptive Geometry, Faculty of Wood Sciences and Technology, Technical University in Zvolen, T. G. Masaryka 24, 96001 Zvolen, Slovakia; schmidtova@is.tuzvo.sk; 6 Institute of Foreign Languages, Technical University in Zvolen, T. G. Masaryka 24, 96001 Zvolen, Slovakia; veverkova@is.tuzvo.sk; 7 Earth Science Institute v.v.i., Slovak Academy of Sciences, Ďumbierska 1, 97411 Banská Bystrica, Slovakia; biron@savbb.sk

**Keywords:** heavy metals, neutral mine drainages, metal adsorption, alkaline treatment, fly ash

## Abstract

Mine effluents represent a serious environmental problem on a global scale. Therefore, the effective treatment of this water is a serious issue in the scientific field. The adsorption process seems to be one of the attractive methods, especially due to the simplicity of design, affordability or high efficiency. The latest scientific knowledge has shown that the use of waste and natural adsorbents is economical and effective. This study aimed to evaluate the efficiency of the adsorption process of natural and waste materials—zeolite, bentonite and fly ash—under the influence of temperature and modification of these adsorbents. The novelty of this study resides in an adjustment of the modification method of adsorbents compared to previous research: thermal–alkaline treatment versus hydrothermal one. Another novelty is the use of modified fly ash from biomass combustion as an adsorbent in comparison with the previously used fly ash from coal combustion. The modification of the adsorbents made the adsorption process more effective at all experimental concentrations. The characterisation of adsorbent samples was performed using X-ray diffraction (XRD). The parameters of the adsorption isotherms, Langmuir, Freundlich and Temkin, were estimated by nonlinear regression analysis. The adsorption capacity of Cu(II) of fly ash was comparable to natural adsorbents. Adsorption processes were better described by pseudo-second-order kinetics. At the end of this study, the suitability of using the adsorbents to reduce the concentration of Cu(II) in neutral mine effluents was observed in the following order at 30 °C: unmodified fly ash > modified bentonite > unmodified zeolite. At the temperatures of 20 °C and 10 °C, the same trend of the suitability of adsorbents use was confirmed: modified bentonite > modified zeolite > modified fly ash. The practical applicability of this study lies in the expansion of knowledge in the field of adsorption processes and in the improvement of waste management efficiency of heating plants not only in Slovakia, but also globally.

## 1. Introduction

Mine wastewater can have very low or, conversely, high pH, depending on complex interactions between hydrological, chemical and biological processes. These waters are typically rich in heavy metals and sulphur (present in minerals) but low in nutrients [1]. Mine drainages also contain chemicals that can pose potential risks, such as salts, nitrates and sulphates [2]. Due to the neutralizing properties of waste minerals or human interventions, such as the addition of limestone to precipitate metals, the pH of these waters can rise to values between 4.5 and 8.5, in which case the water is referred to as neutral mine drainage (NMD) [1].

NMD is less widespread globally than AMD, and since its environmental impacts are not as severe, it has received relatively little attention. Nevertheless, NMD often contains high levels of dissolved metals [3]. NMD is commonly associated with low dissolved oxygen and medium to high concentrations of sulphate and metals that are soluble (e.g., Cu, Zn, Cd) or insoluble (Fe, Al, Mn) under aerobic conditions [4]. Such drainage poses a potentially serious environmental risk because heavy metals, often present at high concentrations, can remain dissolved under suitable redox conditions even in an alkaline environment [1]. Neutral mine drainage (NMD) can pose an environmental risk if the disposal of low-sulphide waste rock at mining sites is not managed properly. Unlike acid mine drainage (AMD), predicting the behaviour of NMD using conventional kinetic tests is challenging, as processes of contaminant immobilization—particularly through sorption and precipitation—occur in these waters [5]. NMD causes surface water contamination, with most cases producing drainage of nearly neutral pH, particularly with elevated zinc concentrations [3].

Excessive levels of the heavy metal Cu(II) in groundwater and surface water can lead to serious environmental contamination [6]. In the site that we monitored, the preliminary measurements in our study revealed increased concentrations of Cu(II) exceeding the limit values. In the human body, copper is essential for the development of connective tissue, nerve sheaths and bones, and it is also a part of several enzymes that catalyse important biological reactions [7].

At present, only limited attention is given to the adsorption of Cu from neutral mine drainage [8,9] compared with its removal from acid mine drainage (AMD) [10,11,12,13,14,15]. Therefore, we decided to focus our study on the removal of ions of this potentially toxic element from NMD. In addition to process efficiency, economic demand is also important in the feasibility of wastewater treatment. Therefore, it is important to consider this factor. Among the costly traditional methods used to remove heavy metal ions from wastewater are chemical precipitation [16], solvent extraction [17] and ion exchange [18]. Adsorption can be considered a favourable alternative for the removal of heavy metal ions from wastewater [19], in which natural materials, agricultural by-products and industrial waste of biological origin can be used as adsorbents of high efficiency [20]. It has been proven that adsorption is an effective cleaning method because it provides significant advantages, including stability, utility, low costs, ease of operation and performance. Adsorption technology has great advantages, since it reduces heavy metal ion concentrations to very low levels and uses various low-cost adsorption materials, including biosorbents, clays, activated carbon, zeolites and metal oxides [21]. Marine red algae [22] and *Saccharomyces cerevisiae* [23] are among the biomaterials recently used in biosorption. We decided to use bentonite and zeolite as natural adsorbents due to their availability. In the past, both materials were confirmed as suitable adsorbents for the treatment of mine effluents. For the treatment of NMD, we also used biomass waste ash, which is available in our location from the heating plant, and also has a positive impact on reducing this waste when used as a secondary product. The positive effect of the fly ash used as a waste adsorbent was confirmed by studies in the past [24,25]. To enhance the adsorption process, thermal–alkaline treatment was employed. The positive effect of the thermal–alkaline treatment on adsorption was confirmed by studies [26,27].

In addition to thermal–alkaline treatment, the effect of temperature change on the sorption course was monitored. Selected studies confirmed the positive effect of temperature on the adsorption of heavy metals [28,29]. However, beyond a certain temperature, a negative effect was confirmed (when the temperature increased) [30]. On the contrary, in the study [31], the direct effect of temperature on improvement or deterioration of adsorption was not confirmed, although the affinity for the binding site decreased significantly with increasing temperature. The main targets of our study are to expand knowledge in the field of adsorption processes (monitoring the effectiveness of adsorption by temperature change, using thermal–alkaline treatment), and to support waste management of heating plants (reducing the volume of waste fly ash by using it as an adsorbent). The practical applicability of this study is that its findings will support the scientific field in the remediation of AMD and the use of waste fly ash as a secondary product, not only in Slovakia but also globally.

The novelty of this study lies in the modification of the adsorbent preparation method compared to previous research, using a thermal–alkaline treatment instead of a hydrothermal one. It has been confirmed that thermal–alkaline treatment leads to an increase in the BET surface area, total pore volume and adsorption capacity of the material [32]. The alkaline activation of the adsorbents was carried out by applying solid pure NaOH and NaNO_3_ (sample ratio, NaOH: NaNO_3_—7:3:10) in an autoclave at a temperature of 350 °C for 12 h. The same methodology was used in the study by Choi et al. [33], but in their case, fly ash was obtained from burning anthracite coal, whereas our fly ash was obtained from burning biomass. The mentioned method of thermal–alkaline treatment in our case differed from hydrothermal–alkaline treatments compared to other studies [34,35]. In the mentioned studies, a highly alkaline environment was created using 5 M NaOH, but the temperature acting on our adsorbents was higher compared to the mentioned studies. In our case, the adsorbents were not introduced into the solution but homogenised with solid alkali. In the study [34], adsorbents were modified at temperatures of 100 and 200 °C during 3–48 h of action, and in the study [35], hydrothermal–alkaline treatment was performed at temperatures of 120–250 °C, during 10–36 h. Unlike the hydrothermal method, our thermal–alkaline pre-treatment method uses a mixture of salts as a reaction medium without any addition of water. The goal of this difference was to monitor the adsorption mechanism under thermal–alkaline pre-treatment conditions without the need to introduce the adsorbent into the solution and to monitor the effect of high temperature on the structure of the adsorbents.

Another innovation is the use of modified fly ash from biomass combustion as an adsorbent, rather than the coal-derived fly ash used earlier. Ash produced from coal and biomass combustion exhibits considerable variability in its physical and chemical properties. Its chemical composition is crucial, as it determines potential applications across different sectors. While coal ash is richer in SiO_2_, Fe_2_O_3_ and Al_2_O_3_, biomass ash contains higher levels of Na_2_O, K_2_O, MgO and P_2_O_5_ [36].

## 2. Materials and Methods

### 2.1. Sampling Locations

**Figure 1:**
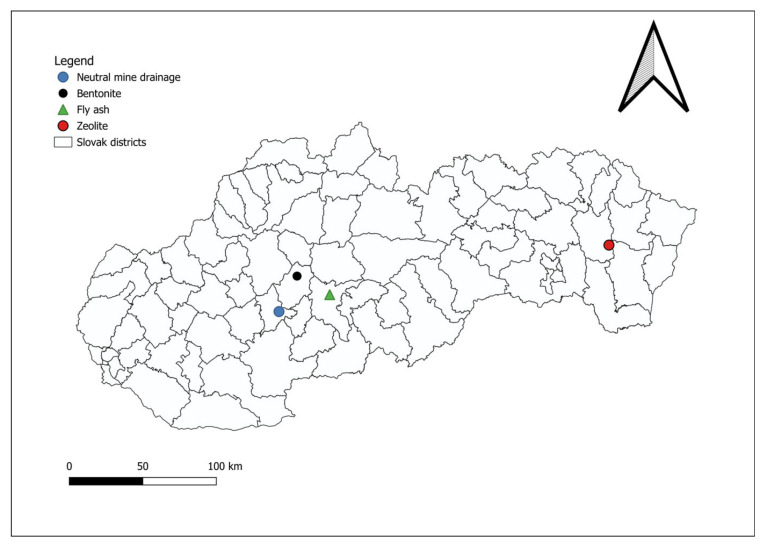
The locations of the sampling sites for the adsorbents and mine discharges.

The neutral mine drainage was collected from the Voznicka heritage tunnel in the Štiavnica–Hodruša mining district (central Slovakia), as shown in Figure 1. Ash from biomass combustion was obtained from the thermal power plant in Zvolen (Figure 1). Natural bentonite was obtained from mining in the Kopernica area (Figure 1). Natural zeolite was obtained from mining in the Nižný Hrabovec area (Figure 1).

Extraction of neutral mine drainage: The neutral mine drainage was taken from the Voznicka heritage tunnel in the Štiavnica–Hodruša mining district (central Slovakia)—48°27′ N latitude and 18°42′ E longitude. This water represents a legacy of mining activities in the area. The total historical production of mines in the mining district is estimated at 4000 t of Ag and 80 t of Au. During the mining of non-ferrous metals (19th century, until 1992), approximately 70,000 t of Zn, 55,000 t of Pb and 8000 t of Cu(II) were mined [37]. The central Slovak volcanic field contains several Ag–Au deposits of the epithermal vein type, which in the past were a source for mining of both precious and base metals [38].

The sampling was carried out at the site where the monitoring of Dionýz Štúr was conducted by the State Geological Institute. The samples were collected in plastic bottles, stored in the cold and transported to the laboratory in accordance with the requirements of STN EN ISO 5667-1:2007 [39]. Guidelines for the design of sampling programmes and sampling techniques. For microbiological analysis, water samples were taken from the heritage drainage tunnels and from drainage water originating in the tailings pond. The samples were collected in sterile glass bottles, kept in the cold and transported to the laboratory in accordance with the requirements of STN EN ISO 19458 [40]. Sampling for microbiological analysis.

### 2.2. Analysis Methods

The individual characteristics of the samples were obtained by standard methods, which are presented in Table 1 and Table 2.

Atomic Absorption Spectrometry (AAS): An AAS AVANTA Σ flame atomisation spectrometer (GBC Scientific) was used to determine metal concentrations during the adsorption process.

Atomic emission spectrometry with the inductively coupled plasma (AES-ICP): A method of emission spectroscopy that excites atoms and ions with plasma, causing them to emit electromagnetic radiation with wavelengths specific to a particular element.

Elemental analysis with thermal conductivity detection (EA-TCD): A thermal conductivity detector (TCD), a katharometer, is a volumetric property detector and a chemical-specific detector commonly used in gas chromatography. This detector detects changes in the thermal conductivity of the eluent of the column and compares it with the reference flow of the carrier gas. Since most compounds have a thermal conductivity much lower than the common carrier gases such as helium or hydrogen, as the analyte elutes from the column, the thermal conductivity of the effluent is reduced, and a detectable signal is produced.

Brunauer–Emmett–Teller (BET) analysis: Multipoint measurement of the specific surface area of an analyte (m^2^·g^−1^) through gas adsorption analysis, where an inert gas such as nitrogen continuously flows through a solid sample, or the solid sample is suspended in a defined volume of gas. Small gas molecules are adsorbed on the solid substrate and its porous structures due to weak Van der Waals forces and form a monolayer of adsorbed gas. This monolayer and adsorption rate can be used to calculate the specific surface area of the solid sample and its porous geometry, providing information for reactivity and bioavailability studies of pharmaceutical products.

Thermal–alkaline activation: In order to modify the adsorbent, thermal–alkaline treatment without the addition of water was used. Typically, mixtures containing 0.7 g of adsorbent, 0.3 g of NaOH, and 1 g of NaNO_3_ were ground to a fine powder in a Pt crucible and heat-treated at 350 °C (±5 °C) for 12 h.

Adsorption of heavy metals: Adsorption was carried out at temperatures of 30 °C, 20 °C and 10 °C in Erlenmeyer flasks. These temperatures were chosen to simulate the climate conditions of the studied area in the spring, summer and autumn periods. The purpose of these temperatures was to better understand the adsorption mechanism under real temperature conditions. The experimental conditions of sorption included stirring at 500 rpm (revolutions per minute) using a magnetic stirrer, a pH of 7.6 and an initial adsorbate concentration (mg·L^−1^) to adsorbent dosage (g) ratio of 20, 36, 48, 64 and 72. During the adsorption, the change of pH in the adsorbates was also monitored. The reason for this is the change in the pH of mine effluents due to specific adsorbents. The sample volume in each flask was 100 cm^−3^. Before adsorption, 500 cm^−3^ of solution was prepared for each input concentration—a total of 5 input concentrations. A volume of 100 cm^−3^ was taken to determine the initial concentration. Then, 100 cm^−3^ of solution was added to each flask and mixed with 0.25 g of adsorbent. After 30, 60, 90 and 120 min, adsorption was stopped by filtering out the adsorbent in individual bottles, and then the concentration of metals—copper—was measured. The temperature was reduced to 10 °C in an incubator. The adsorption of each input concentration was repeated 5 times for the relevance of the evaluation—a total of 6 measurements were performed for each input concentration for each adsorbent at each temperature. The addition of CuSO_4_·5H_2_O was used to change the initial concentrations for Cu adsorption. Adsorption took place under continuous stirring to optimise the contact between the aqueous phase and the adsorbent. Mine water solutions with modified Cu concentrations for the needs of 5 input concentrations were used for adsorption processes.

Determination of pH and oxidation–reduction potential follows from STN EN ISO 10390 using a potentiometrically combined electrode. The oxidation–reduction potential is a measure of electron activity and an indicator of the ability of a biogeochemical system to receive or transfer electrons. It is the electrical potential required to transfer electrons from one compound/element (oxidising agent) to another compound/element (reducing agent). Oxidation–reduction potential values close to −300 mV indicate a highly reducing environment or the ability to supply electrons, while values from +100 mV to +300 mV indicate a highly oxidising environment or the ability to accept electrons [46]. The inoLab pH Level 1 device (WTW Weilheim, Weilheim in Oberbayern, Germany) was used to determine pH and oxidation–reduction potential.

X-ray powder diffraction: Mineralogical composition of both initial and treated samples was determined using the X-ray powder diffraction technique. Sample preparation for XRPD analysis was conducted as follows. Exactly 0.5000 g of internal standard Al_2_O_3_ (AL-OX-03-P, nominal grain-size 3–4 µm; producer: American Elements Corp., Los Angeles, CA, USA) was added to 2.0000 g of sample mixed with 4 cm^−3^ of ethanol and ground for 5 min in McCrone Micronising Mill (©Retsch GmbH, Haan, Germany) using a set of zirconia grinding elements. The resulting slurry was dried at 75 °C overnight. To achieve maximum randomisation of the sample, water slurry was prepared and spray-dried using the Spray Drying Kit (The James Hutton Institute, Aberdeen, Scotland [47]).

The XRPD analyses were conducted on a Bruker D8 Advance diffractometer (Karlsruhe, Germany) using β-filtered CuKα radiation generated at 40 kV and 40 mA and a position-sensitive SSD160 detector working in the 1D regime. The beam was collimated with a slit assembly 0.3–6 mm PSD opening 0.4984°, with primary and secondary Soller slits 2.5°. Diffraction patterns were recorded from 3–70°2θ with a step size of 0.01948°2θ and counting 2s per step. Samples were analysed in a top-loaded holder.

The evaluation of XRPD patterns and the identification of individual phases were carried out with DIFFRAC.EVA software Version 4.2.1.10 [48]. The quantitative Rietveld analysis was performed with TOPAS 5 software using a fundamental parameters approach (internal standard method) [49,50]. As starting structural models, the following published data were used: McGinnety (1972) for arcanite [51], Hassan & Grundy (1991) for cancrinite [52], Comodi et al. (2004) [53] for biotite (phlogopite), Hughes et al. (1989) for hydroxylapatite [54], Angel et al. (2013) for K-feldspar (orthoclase) [55], Hazen (1976) for periclase [56], Gualtieri (2000) for plagioclase [57], Desgranges et al. (1993) for portlandite [58], Antao et al. (2008) for quartz [59] and Gournis et al. (2008) for smectite (montmorillonite) [60]. During the Rietveld refinement, the following parameters were considered: emission profile of radiation, scale factor, polynomial background, correction of the zero-point of the goniometer, sample displacement error, lattice parameters and atomic positions (major phases only). However, atomic occupancy and isotropic displacement factors were kept fixed. No attempt to model the turbostratic disorder common for smectite-group phyllosilicates was made.

Samples revealing high amounts of smectite or clinoptilolite were quantitatively evaluated with RockJock Version 1111 software (method based on full-pattern summation and mineral intensity factors [61,62]). The mineral composition of initial samples/materials and its changes upon thermochemical treatment is shown in Table 3.

The powder X-ray diffraction analysis of adsorbent samples (Table 3) before adsorption experiments confirmed the change in the mineralogical structure of the adsorbents after the alkaline modification process.

**Figure 2:**
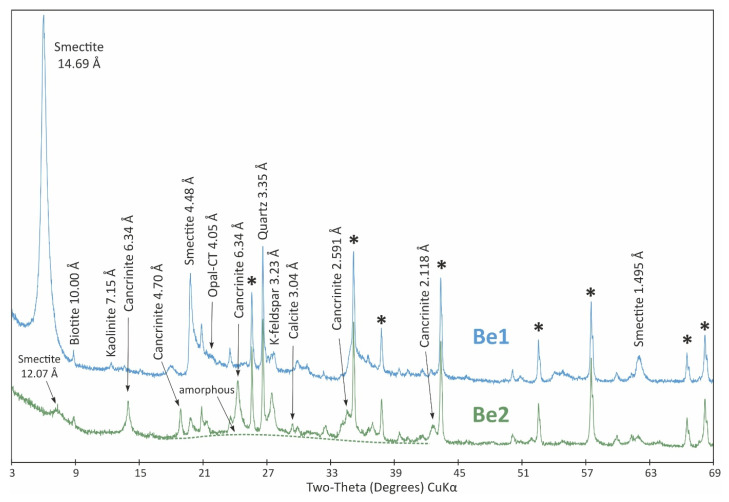
XRPD patterns of initial (Be1) and treated (Be2) bentonite (Kopernica deposit). *—internal standard: corundum. The dashed line schematically depicts the amorphous component.

**Figure 3:**
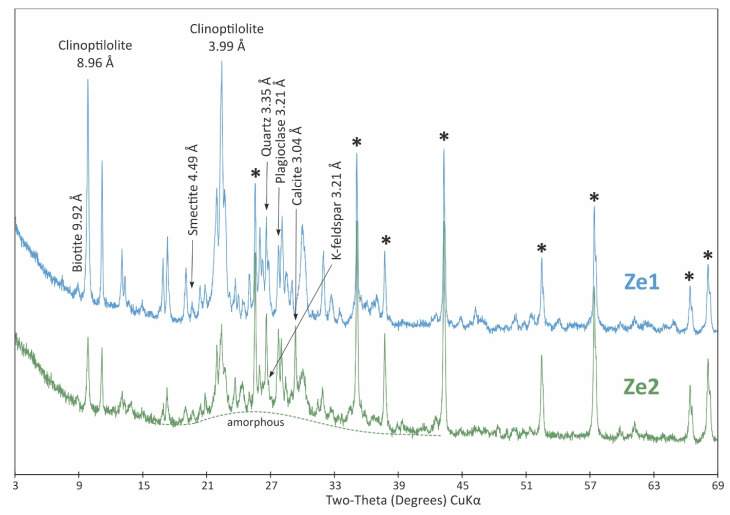
XRPD patterns of initial (Ze1) and treated (Ze2) zeolite (Nižný Hrabovec deposit). *—internal standard: corundum. The dashed line schematically depicts the amorphous component.

**Figure 4:**
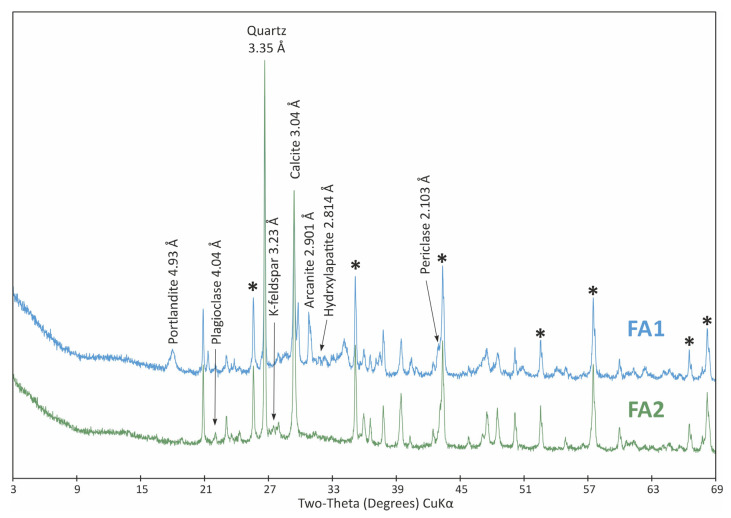
XRPD patterns of initial (FA1) and treated (FA2) fly ash. *—internal standard: corundum.

The XRPD patterns of initial and treated adsorbents are shown in Figure 2, Figure 3 and Figure 4.

The XRPD pattern of original bentonite, shown in Figure 2 (Be1), indicated dioctahedral smectite (d(06,33) = 1.495 Å) with minor admixtures of K-feldspars, quartz, opal-CT, biotite and kaolinite. The quantitative analysis revealed the presence of 73 wt.% smectite, 11 wt.% K-feldspars (orthoclase and sanidine), 6 wt.% quartz and opal-CT and 2 wt.% biotite and kaolinite. The XRPD pattern of the treated sample (Figure 2, Be2) displayed a significant decrease in the intensity of both basal and non-basal smectite reflections. The decrease in basal spacing from 14.7 Å to 12.1 Å indicates changes in the interlayer cation composition and hydration state (i.e., divalent cations were replaced by monovalent ones). Quantitatively, smectite formed only app. 1 wt.% of the treated sample, and it was replaced by the newly crystallized phase, identified as cancrinite (16 wt.%). Traces of calcite (<1 wt.%) were also recorded. Minor phases do not show significant quantitative changes except for the disappearance of opal-CT and kaolinite. However, it should be noted that in treated bentonite, the most abundant reaction product is the amorphous component (72 wt.%, possibly silica). This is documented on the XRPD pattern by an elevated background in the interval 19–35°2θ.

The XRPD analysis of zeolite from the Nižný Hrabovec deposit showed dominancy of clinoptilolite, which formed 76 wt.% of the sample (Figure 3). Supplementary phases are represented by minor opal-CT (15 wt.%) and accessory plagioclase (4 wt.%), smectite (3 wt.%), biotite and K-feldspar (both <1 wt.%). The treatment resulted in a reduction in clinoptilolite content to 29 wt.%, an increase in plagioclase content to 11 wt.% and the formation of 4 wt.% calcite (Figure 3). Again, the most pronounced response to the treatment procedure was the appearance of 50 wt.% of amorphous material.

The XRPD analysis of original ash allowed the identification of several mineral phases, which were subsequently completely removed from the sample after thermochemical treatment (Figure 4, Po1): arcanite (17 wt.%), portlandite (16 wt.%), periclase (6 wt.%) and hydroxylapatite (5 wt.%). Instead, 35 wt.% plagioclase and 7 wt.% K-feldspar were identified as reaction products (Figure 4, Po2). On the other hand, only minor increases in calcite (from 29 to 34 wt.%) and quartz (from 22 to 24 wt.%) contents were detected.

In zeolite, clinoptilolite (76 wt.%) and opal-CT were initially dominant. After modification, the clinoptilolite content decreased to 29 wt.%, the amount of plagioclase increased to 11 wt.%, and calcite formed at 4 wt.%. These structures are favourable for the incorporation of Cu(II) into the newly formed minerals. The most significant response to the modification procedure was the occurrence of 50 wt.% amorphous material, which likely limited the higher adsorption capacity of the modified zeolite. A temperature of 20 °C appears to be the most favourable for adsorption by the modified zeolite.

The modification processes of both bentonite and zeolite altered the mineralogical composition of their phases. In bentonite, opal-CT and kaolinite disappeared, and there was a significant decrease in the intensity of both basal and non-basal reflections of smectite. These minerals were replaced by a newly crystallized cancrinite phase (16 wt.%), which could effectively immobilize Cu(II) in the final matrices enriched with cancrinite. The immobilization mechanism involves the speciation of silicates, hydroxides or oxides and the encapsulation of such substances within cancrinite agglomerates and other aluminosilicate agglomerates. The lower BET surface area is likely due to the presence of the amorphous component.

The behaviour of fly ash is related to the mineralogical changes occurring during its thermal modification. While the raw fly ash contained 44 wt.% mineral phases such as arcanite, portlandite, periclase and hydroxylapatite, in the modified fly ash, the disappearance of these phases was accompanied by an increase in calcite and, especially, plagioclase content. The adsorption of Cu(II) in fly ash at relatively high pH (9.2–9.75) was likely influenced by the presence of periclase and portlandite, and elevated temperature promoted surface reaction mechanisms on the adsorbent.

In the case of modified fly ash, the increased content of plagioclase and calcite contributed to the immobilization of Cu(II) through its incorporation into the structures of aluminosilicate agglomerates. At 30 °C, desorption processes likely occurred due to the increased heterogeneous surface area.

Most of the identified mineral phases are sodium/calcium aluminosilicates (cancrinite, smectite and plagioclase). In the amorphous structure of modified zeolites and bentonites, the negative charge does not appear to be localized but is more or less evenly distributed within the structure. The Si(IV) and Al(III) cations in the aluminosilicate framework are tetrahedrally coordinated and interconnected by oxygen bridges. The negative charge on the AlO_4_^−^ group is balanced by alkali cations (typically Na^+^ or K^+^). Based on XRD analysis, we identified the presence of an amorphous Al-Si-O structure, which is also responsible for the adsorption of the tested copper ions. The presence of the amorphous component enhances the adsorption properties of the materials [63,64].

Microscopic images of crystal size: measured using a VHX-7000 digital microscope (Keyence, Osaka, Japan). Microscopy was performed on different types of fractions: bentonite (1.2–2 mm); modified bentonite (0.3–0.5 mm); fly ash and modified fly ash (0.1–0.5 mm); zeolite and modified zeolite (0.5–1 mm). The fly ash fraction was obtained in its original form (without crushing). Natural bentonite was crushed to bring its grain size closer to that of the other adsorbents used. The zeolite fraction was not adjusted, as the particles already in these sizes approached the size of bentonite.

**Figure 5:**
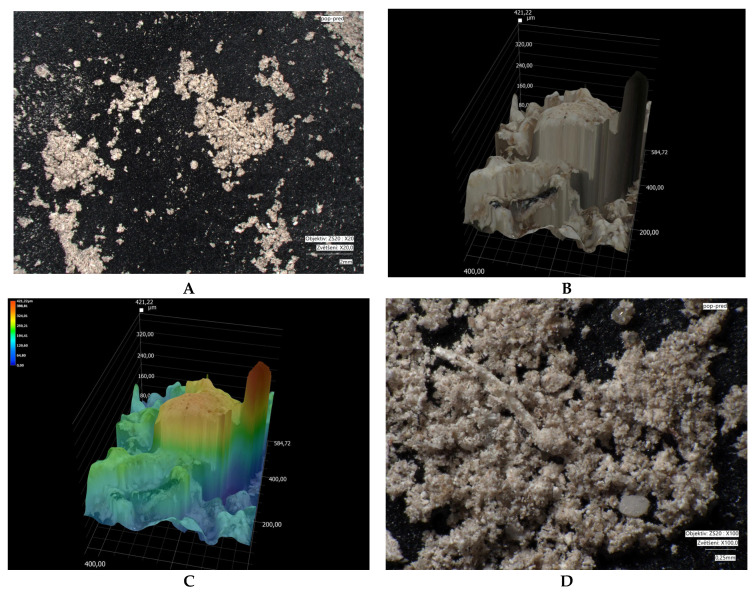
Fly ash, before treatment: (**A**) sorbent grain size at 20-fold magnification; (**B**) surface structure in 3D, with a max depth of 245 μm; (**C**) colour resolution of surface irregularities; (**D**) microstructure at 100-fold magnification.

**Figure 6:**
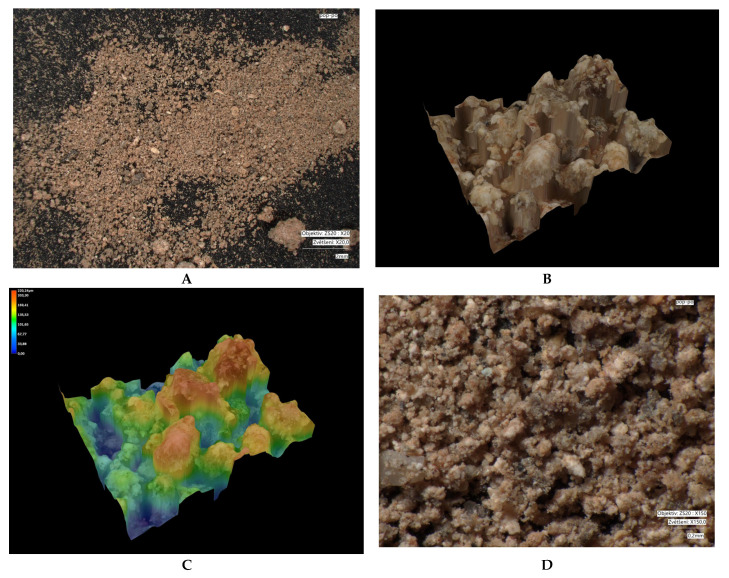
Fly ash, after treatment: (**A**) grain size of the sorbent at 20-fold magnification; (**B**) surface structure in 3D with a max depth of 220 μm; (**C**) colour resolution of surface irregularities; (**D**) microstructure at 150-fold magnification.

**Figure 7:**
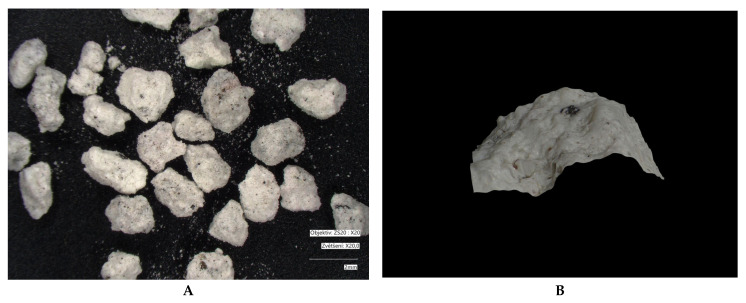

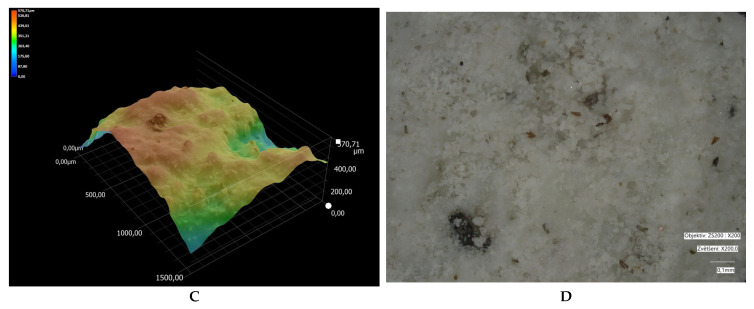
Bentonite, before treatment: (**A**) grain size of the sorbent at 20-fold magnification; (**B**) surface structure in 3D, with a max depth of 570 μm; (**C**) colour resolution of surface irregularities; (**D**) microstructure at 200-fold magnification.

**Figure 8:**
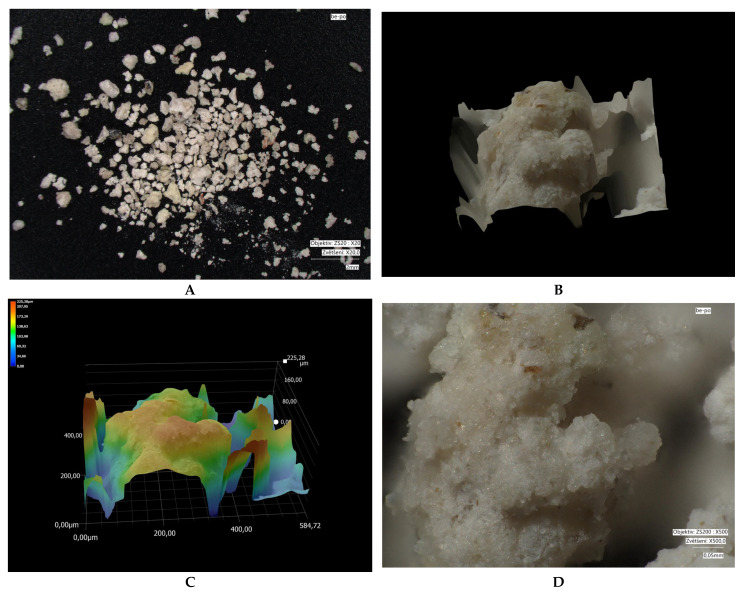
Bentonite, after treatment: (**A**) grain size of the sorbent at 20-fold magnification; (**B**) surface structure in 3D, with a max depth of 225 μm; (**C**) colour resolution of surface irregularities; (**D**) microstructure at 500-fold magnification.

**Figure 9:**
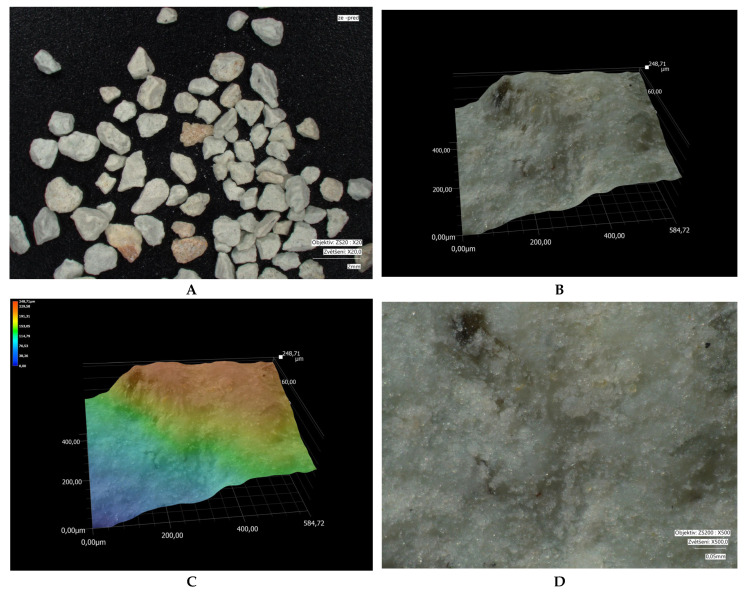
Zeolite, before treatment: (**A**) sorbent grain size at 20-fold magnification; (**B**) surface structure in 3D, with a max depth of 249 μm; (**C**) colour resolution of surface irregularities; (**D**) microstructure at 500-fold magnification.

**Figure 10:**
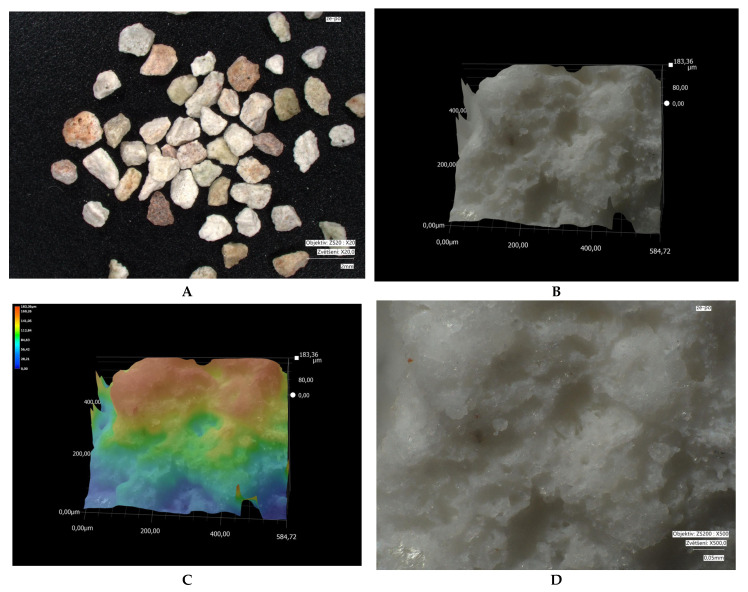
Zeolite, after treatment: (**A**) sorbent grain size at 20-fold magnification; (**B**) surface structure in 3D, with a max depth of 183 μm; (**C**) colour resolution of surface irregularities; (**D**) microstructure at 500-fold magnification.

Microscopic analyses are presented in Figure 5, Figure 6, Figure 7, Figure 8, Figure 9 and Figure 10. Microscopic analysis was used to improve knowledge about the structural properties of sorbents.

Figure 5 and Figure 6 illustrate the changes in the microstructure of fly ash before and after thermal treatment. After modification, the particle size of the fly ash decreased, which was also reflected in an increased BET surface area. The surface became more consistent and compact. Part B of the images highlights the formation of a multilayered heterogeneous surface after fly ash modification.

Figure 7 and Figure 8 show that in bentonite, similar to fly ash, grain fragmentation and disruption of the surface structure occur; however, this does not compromise the specific surface area of the modified bentonite adsorbent.

Figure 9 and Figure 10 indicate that thermal treatment of zeolite did not significantly alter its particle size, nor was the adsorbent’s morphology substantially changed. Part D of the images demonstrates that the zeolite surface was consolidated during modification (losing its porous structure), which is also evidenced by the reduction in specific surface area measured by BET analysis.

### 2.3. Calculations

#### 2.3.1. Freundlich, Langmuir and Temkin Adsorption Isotherms

To express the dependence of the amount of adsorbed metal ion on its equilibrium concentration in the solution, the Freundlich and Langmuir isotherms were constructed for all adsorbents used. The isotherms were evaluated at five initial concentrations.

#### 2.3.2. Freundlich Adsorption Isotherm

The Freundlich isotherm is usually valid for physical adsorption and for adsorption on heterogeneous surfaces with different active sites. It can be expressed by the formula:

(1) $$q_{e}=K_{f}\cdot c_{e}^{\;\frac{1}{n}}$$

To verify that the experimental data satisfy this isotherm, the relationship is linearised:

(2) $${log\;q}_{e}={log\;K}_{f}+\frac{1}{n}{log\;c}_{e}$$

#### 2.3.3. Langmuir Adsorption Isotherm

The Langmuir isotherm usually applies to chemisorption or electrostatic adsorption, in which only a monomolecular layer is formed on the surface of the adsorbent, and all active centres are equivalent. The Langmuir isotherm is expressed by the formula

(3) $$q_{e}=\frac{q_{m}\cdot b\cdot c_{e}}{1+b\cdot c_{e}}\;$$

Or by the linearised formula according to Hanes–Wolf:

(4) $$\frac{c_{e}}{q_{e}}=\frac{1}{b\cdot q_{m}}+\frac{1}{q_{m}}\cdot c_{e}\;$$

The Langmuir constant b (L/mg) is used to calculate RL, the dimensionless separation factor, which is given by the equation [65,66]

(5) $$R_{L}=\frac{1}{1+b\cdot C_{o}}\;$$

K_R_ values indicate whether adsorption is unfavourable (RL > 1), linear (RL = 1), favourable (0 < R_L_ < 1) or irreversible (R_L_ = 0) [67].

#### 2.3.4. Temkin Adsorption Isotherm

According to the Temkin isotherm, the heat of sorption should decrease linearly with sorption coverage on the adsorbent due to interactions between the adsorbent and the adsorbate [68,69]. The Temkin isotherm equation is expressed as follows:

(6) $$q_{e}=\frac{R\cdot T}{b_{T}}\;(\ln\;K_{T}\;\cdot c_{e})\;$$

Equation (8) can be presented in the linear form as follows [70,71]:

(7) $$q_{e}=\frac{R\cdot T}{b_{T}}\;\ln\;K_{T}\;+\;(\frac{R\cdot T}{b_{T}}\;\ln\;c_{e})\;$$

where 

K_T_ (dm^3^·g^−1^) is the equilibrium binding energy, which corresponds to the optimum binding energy; b_T_ (J·mol^−1^) is a constant, which is related to the heat of adsorption;

R is the ideal gas constant;

T is the absolute temperature.

#### 2.3.5. Adsorption Capacity

From the measured concentrations, the adsorption capacity in the equilibrium state (q_e_), the amount of metal adsorbed per unit of adsorbent at time t (q_t_) and the percentage removal efficiency of Cu(II) ions from the solution (Ads. %) were calculated. The adsorption capacity at equilibrium and at time t was calculated according to the equation:

(8) $$q_{e}=\frac{\left(c_{o}-c_{e}\right)\cdot V}{m}$$

where the variable q_e_ represents the adsorption capacity at equilibrium and is expressed in milligrams of adsorbed substance per gram of adsorbent (mg·g^−1^). It quantifies the amount of heavy metal that binds to one gram of the adsorbent once the system reaches equilibrium, meaning that the concentration in the solution no longer changes significantly. The initial concentration of the substance in the solution, denoted as c_o_ and measured in milligrams per cubic decimetre (mg·dm^−3^), refers to the concentration of the ionic species present before the adsorption process begins, at time zero. The equilibrium concentration c_e_, also in mg·dm^−3^, is the concentration of the substance remaining in the solution after adsorption has occurred, and equilibrium has been established, i.e., when the concentration remains stable or changes insignificantly over time. The volume of the solution V, expressed in cubic decimetres (dm^3^), indicates the total volume of the solution in which adsorption takes place. Lastly, the variable m stands for the mass of the adsorbent added to the solution, measured in grams (g), which is the amount of material used to remove the targeted substance from the solution.

Experiments focused on copper adsorption were carried out with natural, unmodified adsorbents in a closed system with constant stirring of the suspension at room temperature. We monitored the course of sorption depending on the adsorbent used.

#### 2.3.6. Kinetics of Sorption

We used two kinetic models to describe the adsorption kinetics: the pseudo-first-order model [72] and the pseudo-second-order model [73]. The pseudo-first-order model assumes that the rate of metal adsorption is directly proportional to the number of unoccupied binding sites on the surface of the adsorbent. The pseudo-second-order model assumes that the rate of metal adsorption is directly proportional to the square of the remaining binding sites.

Pseudo-first-order equation:

(9) $$\frac{dq_{t}}{dt}=k_{1}\left(q_{e}-q_{t}\right)$$

After adjustment, including the separation of variables, integration and substitution of boundary conditions (t = 0 to t; q_t_ = 0 to q_e_), the equation takes the following form:

(10) $$\ln\left(q_{e}-q_{t}\right)=\;k_{1}(q_{e}-\;q_{t})$$

and the rate constant k_1_ is determined from the linear dependence guideline:

(11) $$\ln\left(q_{e}-q_{t}\right)=\;f(t)$$

The rate equation for the pseudo-second-order kinetic model is expressed by the formula

(12) $$\frac{dq_{t}}{dt}=k_{2}{\left(q_{e}-q_{t}\right)}^{2}$$

After adjustment, including separation of variables, integration and substitution of boundary conditions (t = 0 to t; q_t_ = 0 to q_e_), the equation takes the following form:

(13) $$\frac{t}{q_{t}}=\frac{1}{k_{2}.q_{e}^{2}}+\frac{1}{q_{e}}t$$

The dependence t/q_t_ = f (t) is linear, and the values of q_e_ (from the direction) and k_2_ (from the section on the y-axis) can be calculated from it.

#### 2.3.7. Thermodynamic Quantities

For the adsorption of copper on bentonites, we also calculated the values of thermodynamic variables ΔH^0^, ΔS^0^ and ΔG^0^ according to

(14) $$lnK_{C}=\frac{∆S^{0}}{R}-\frac{∆H^{0}}{R\text{.}T}$$

(15) $$∆G=-R.T.lnK_{c}$$

From the linear dependence ln K_c_ = f(1/T), we determined the values of ΔH^0^ (from the direction of the straight line) and ΔS^0^ (from the section cut by the straight line on the y-axis).

### 2.4. Statistical Analysis

In addition to descriptive characteristics, selected methods of statistical inference were applied in the statistical processing of data.

Linear and nonlinear regression methods were used to model the investigated dependencies. The statistical significance of the created models was subsequently tested using the F-test. The F-statistic test represents the ratio of two variance estimates—the variance estimate explained by the regression model (MS effect) and the residual variance estimate (MS error). If the MS effect is significantly larger than the MS error, the regression model is suitable for the mathematical definition of the observed dependency. When evaluating other areas of research (adsorption capacities, pH), interval estimates of population means with a 95% confidence level were used. Some of them are illustrated using box plots. All analyses were performed using STATISTICA 14 statistical software. The alpha level of 0.05, traditionally used in similar studies as a decision rule, was applied. The output tables were edited in the Microsoft Excel spreadsheet editor for better clarity [74].

## 3. Results

### 3.1. Characteristics of Neutral Mine Drainage

An increased concentration of Cu was measured in the water from the Voznicka heritage tunnel of 17.2 µg·dm^−3^, which is the limit value for all hardness classes (Table 4). The mining discharges at the monitored site are of a specific neutral nature, which is unusual given their mining origin.

Among all the monitored metals, copper (Cu) stands out, with a concentration of 17.2 µg·dm^−3^ in the sample, significantly exceeding the limits for water hardness classes 1 and 2 (1.1 µg·dm^−3^) and for classes 4 and 5 (8.8 µg·dm^−3^) (Table 4). Other metal elements, including Ag, Ba, Cd, Co, As, Pb, Se, Sb, Sn, Sr, Fe, Zn, Tl and Al, are either within permissible limits or their concentrations do not pose an environmental risk. The sample has a neutral pH (7.6), confirming that the exceedance of the copper limit is not associated with extreme acidity or alkalinity. This result indicates that Cu represents the most significant environmental concern in the analysed NMD water and requires targeted measures for its reduction.

### 3.2. Characteristics of Natural Sorbents

The composition of the adsorbents used is presented in Table 5.

The chemical composition indicates that SiO_2_ is the dominant component in both sorbents, with bentonite containing 60.18–73.86% and zeolite 64.18–75.50%. The Al_2_O_3_ content ranges from 11.56 to 24.90% in bentonite and from 10.93 to 14.80% in zeolite. Fe_2_O_3_ is higher in bentonite (2.15–3.39%) than in zeolite (0.12–2.45%). Other oxides present include CaO, MgO, TiO_2_, Na_2_O, K_2_O and MnO, with concentrations varying between the sorbents, reflecting differences in mineral structure and potential adsorption capacity. Zeolite contains higher amounts of CaO and K_2_O, whereas bentonite has higher Al_2_O_3_ and Fe_2_O_3_ contents.

Elemental analysis shows that bentonite contains more aluminium (121,198 mg·kg^−1^). The contents of toxic elements such as Pb, Cd and As are low in both sorbents, with bentonite having slightly higher levels of lead (12.4 mg·kg^−1^) and arsenic (6.3 mg·kg^−1^) compared to zeolite. These data suggest that both sorbents possess suitable chemical properties for adsorption, while the differences in mineral composition may influence their selectivity toward various ions.

### 3.3. Characteristics of Fly Ash

The ash was produced by burning wood chips (biomass). This is the finest ash captured by the electrofilter before it enters the chimney (Table 6 and Table 7). The properties of the biomass combustion ash used for Cu adsorption are shown in Table 6 and Table 7.

The fly ash used in this study has a very high dry matter content of 99.97%, indicating that it is practically completely dry. It is strongly alkaline, with a pH of 12.11, which may affect its chemical reactivity and ion mobility in the environment.

Table 7 presents the BET surface area of the sorbents used in this study, expressed in m^2^·g^−1^. Among the natural sorbents, bentonite has the largest surface area (43.10 m^2^·g^−1^), followed by zeolite (33.51 m^2^·g^−1^) and fly ash (15.43 m^2^·g^−1^). After modification, the surface areas of most sorbents changed: the surface area of modified fly ash slightly increased to 16.45 m^2^·g^−1^, while the surface area of modified bentonite decreased to 27.47 m^2^·g^−1^. The largest reduction was observed for the modified zeolite, whose surface area dropped to 10.10 m^2^·g^−1^. These differences indicate that the modification procedures affect the structure and availability of surface active sites of the sorbents, which can have a significant impact on their adsorption properties.

### 3.4. Nonlinear Regression Analysis, Nonlinear Adsorption Isotherm Models

To express the dependence of the adsorbed amount of the metal ion on its equilibrium concentration in the solution, we constructed the Freundlich, Langmuir and Temkin isotherms for Cu(II) within the concentration range from 5 to 18 mg·dm^−3^ for all the adsorbents used (Table 8).

The equilibrium concentrations of Cu^2+^ at different temperatures were fitted with nonlinear curves of three isothermal models. Temperature had a positive effect on adsorption by fly ash: with increasing temperature, the adsorption capacity rose, reaching a maximum Langmuir capacity (q_m_) of 36.467 mg·g^−1^ at 30 °C. For both fly ash and modified fly ash, the Freundlich heterogeneity parameter (1/n) fell within 1 < n < 10, indicating favourable adsorption. The highest n value was observed for modified fly ash at 30 °C, suggesting increased surface heterogeneity after thermal–alkaline treatment.

Among the tested adsorbents, the highest q_m_ was obtained for fly ash (36.467 mg·g^−1^, 30 °C), followed by modified bentonite (34.109 mg·g^−1^, 30 °C) and zeolite (22.162 mg·g^−1^, 30 °C). The tThermal–alkaline treatment of fly ash improved q_m_ at 10 and 20 °C but decreased it at 30 °C, likely due to enhanced desorption. The Temkin model showed a strong increase in sorption heat at 30 °C: the untreated fly ash released 694.579 J·mol^−1^, while the modified fly ash released 3975.660 J·mol^−1^, indicating a transition from physical to chemical adsorption.

For bentonite, alkaline treatment substantially increased adsorption at all temperatures. At 30 °C, q_m_ rose from 7.541 to 34.109 mg·g^−1^, accompanied by higher sorption heat (1523.130 vs. 3289.896 J·mol^−1^), again pointing to a shift toward chemisorption. The Freundlich constant n ranged from 1.447 to 4.970, with the Langmuir model providing the best fit for untreated bentonite, while the Freundlich model better described modified bentonite.

Zeolite showed lower adsorption than fly ash or bentonite. The Freundlich n values (1.316–2.194) confirmed favourable, though weaker, adsorption. Alkaline treatment enhanced capacity at 20 °C, more than doubling q_m_, but reducing it at 30 °C. For zeolite, the Temkin model provided the best fit, while modified zeolite at 20 °C was better described by the Freundlich model. Sorption heat varied little with temperature: 998.073–1152.540 J·mol^−1^ for untreated and 708.488–1046.620 J·mol^−1^ for modified zeolite, indicating only minor thermal effects.

The separation factor is an important indicator for determining the favourability of adsorption (Table 9).

The R_L_ values indicate whether the adsorption is favourable (0 < R_L_ < 1), unfavourable (R_L_ > 1), linear (R_L_ = 1) or irreversible (R_L_ = 0) [75]. The R_L_ values at concentrations of 5–18 mg dm^−3^ Cu(II) for the sorbents used indicate that the Langmuir model is favourable, as shown by 0 < R_L_ < 1; however, the Freundlich model also implies favourability, as indicated by 0.1 < 1/n < 1.

The isotherms of adsorbents in relation to temperature before and after treatment are shown in Supporting Information, Figures S1–S27.

The statistical characteristics of the isotherms depending on the adsorption conditions are presented in Table 10.

### 3.5. Models of Pseudo-First-Order and Pseudo-Second-Order Kinetics

The models of pseudo-first-order and pseudo-second-order kinetics are indicated in Table 11.

The rate constant of PSO *k*_2_ is used to describe the rate of adsorption equilibrium. In most observations, the speed increased with rising temperature. The highest achieved values were monitored at the highest initial concentration of the adsorbate (18 mg·dm^−3^) with modified fly ash at 10 and 20 °C: 54.778 and 63.965 g·mg^−1^·min^−1^ (Table 11). Modified fly ash had the highest value of this parameter among all adsorbents tested.

A slight effect of the temperature was observed at higher initial concentrations of adsorbate, i.e., at 12, 16, and 18 mg·dm^−3^ and unmodified adsorbents. The values of the rate constant for modified zeolite showed a decrease with rising temperature at all initial concentrations except for the highest one of 18 mg·dm^−3^.

The modification of the adsorbents had a clearly positive effect on the adsorption rate, as described by the PSO model. At all initial concentrations, higher adsorption rates were observed for the modified adsorbents compared with their unmodified forms.

### 3.6. Thermodynamic Parameters

The evaluation of thermodynamic parameters was performed to better understand the adsorption mechanism. The thermodynamic parameter values at three different adsorption temperatures for the three different adsorbents and their modified forms are summarised in Table 12.

All the results of the free Gibbs energy have a negative value, which indicates a spontaneous adsorption process. The more negative the value of ∆G°, the more energy-efficient the process. For all adsorbents, an increase in adsorption temperature has a positive effect, with the ∆G° values becoming more negative. The effect of thermal–alkaline treatment was likewise positive compared with the untreated equivalents of the adsorbents.

### 3.7. Adsorption Capacities

Monitoring the adsorption capacities is necessary from the point of view of the overall course of adsorption (Table 13). It is important to identify when adsorption begins, or at what time the equilibrium of the adsorption process occurs. Descriptive statistics of the adsorption capacity are presented in Table 13.

At temperatures of 20, 40 and 60 °C, adsorption capacities of approximately 5, 5.6 and 6.4 mg·g^−1^ were observed at an initial concentration of 3 mg·dm^−3^. Differences from our study may be caused by the nature of the sorbed medium. In our study, a neutral mine effluent containing other metals was used. In our study, at a similar concentration, higher adsorption capacity was achieved at 10 °C than in the reported study, so the subsequent increase in adsorption capacity as temperatures increased was less significant.

By evaluating the adsorption capacity of fly ash in terms of the influence of temperature, an increase in this indicator due to increasing temperature was found. At the same time, it was observed that the effect of temperature on the increased adsorption capacity of fly ash was more noticeable at higher input concentrations (12, 16 and 18 mg·dm^−3^) than at lower input concentrations (5 and 9 mg·dm^−3^). At the initial concentration of 18 mg·dm^−3^, an increase in adsorption capacity was observed at 10, 20 and 30 °C averaging 6.654, 7.123 and 7.067 mg·g^−1^.

### 3.8. Effect of pH on the Adsorption Process

**Figure 11:**
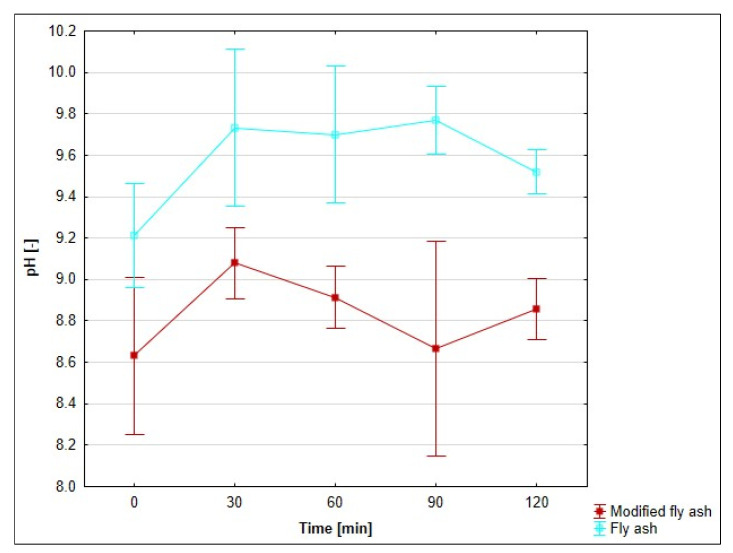
Course of pH depending on the temperature of fly ash.

**Figure 12:**
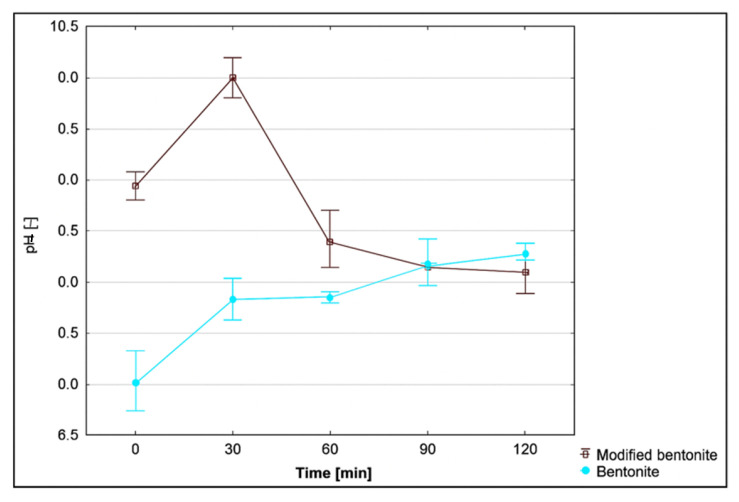
Course of pH depending on the temperature of bentonite.

**Figure 13:**
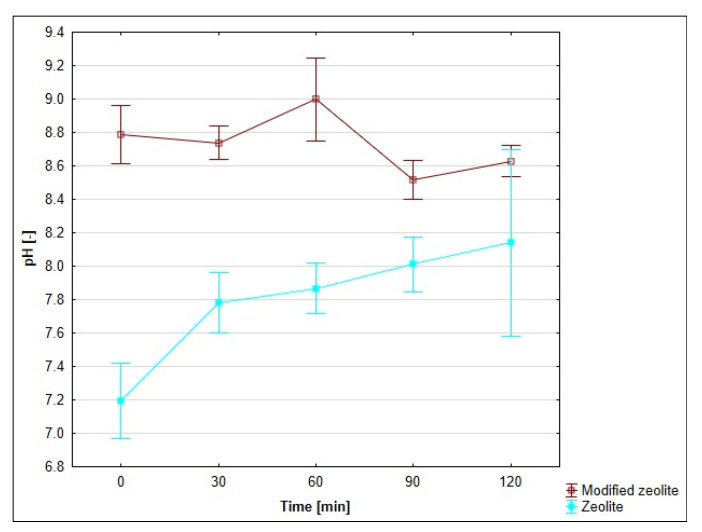
Course of pH depending on the temperature of zeolite.

pH is a necessary indicator for adsorption monitoring. For this reason, pH was evaluated as a function of the duration of adsorption. The course of pH for individual adsorbents, depending on time, is presented in Figure 11, Figure 12 and Figure 13. The pH monitoring was carried out at a medium temperature of 20 °C, as there is no assumption of significant differences in pH depending on temperature.

The pH of the drainage was neutral, with a value of 7.35. The pH values of all three monitored adsorbents and their modified forms were measured after moistening with 100 cm^3^ of original mine water. The values of pH adsorbates with unmodified bentonite and zeolite decreased slightly at time 0 (by 2.7 and 1%). During the following periods of time, they rose at a moderate pace: bentonite by 12.9% and zeolite by 8.7%. Fly ash is alkaline in nature, and its insertion into neutral mine water sharply increased the pH value of the adsorbate to 9.32 (by 26.8%). During the next half-hour measurements, it oscillated around the value of 9.7.

The zeolithization of bentonite increased the pH of the mine water solution compared to unmodified bentonite, with the greatest effect observed after 30 min of stirring: an increase of 2.16 pH units (27.6%). Other half-hour samples showed a decrease in pH differences. In the case of modified zeolite, similar to the modified bentonite, higher pH values were measured, compared to the unmodified form. The largest pH difference was recorded at time 0; in other half-hour samples, we observed a steady-state difference in pH values. The difference was in the range of ⟨0.49–0.89⟩. The modified fly ash behaved differently. Due to the unmodified form of fly ash, the pH values were lower and stabilised at an average difference of 0.74 pH units.

## 4. Discussion

### 4.1. Evaluation of the Nonlinear Correlation of Adsorption Isotherms

In any single-component isotherm study, determining the most appropriate model is key for the mathematical description of the respective sorption system. According to the study by Vitek, Masini [76], nonlinear regression analysis should be preferred over linearised equations (Hanes–Wolf, Lineweaver–Burk, Eadie–Hoffsiee, Scatchard equations) for the accurate characterization of adsorption capacities and adsorption affinities. The use of nonlinear procedures for the estimation of adsorption isotherm parameters increases the quality of the obtained results.

### 4.2. Evaluation of the Parameters of Adsorption Isotherms

The adsorption process forms a layer of adsorbate (metal ions) on the surface of the adsorbents. Adsorption can be reproduced for multiple applications through a desorption method (reverse adsorption, in which adsorbed ions are transported from the adsorbent surface), as adsorption is, under certain conditions, a reversible process. Adsorption onto a solid adsorbent involves three main steps: the transport of the contaminant to the adsorbent surface from the aqueous solution (external diffusion), adsorption onto the solid surface and transport within the adsorbent particle (internal diffusion). In general, electrostatic attraction drives the adsorption of charged contaminants onto differently charged adsorbents, as heavy metals have a strong affinity for hydroxyl (OH^−^) or other functional groups on surfaces.

Adsorption is generally divided into two types: physical adsorption and chemisorption (also described as activated adsorption). Physical adsorption is the adhesion of the adsorbate to the adsorbent surface due to non-specific (i.e., independent of material type) van der Waals forces, whereas chemisorption occurs when chemical bonds create strong attractive forces; that is, chemical adsorption forms ionic or covalent bonds through chemical reactions. Physical adsorption is reversible but less specific, while chemisorption is irreversible but more specific.

Physical adsorption, chemisorption, electrostatic interactions, simple diffusion, intraparticle diffusion, hydrogen bonding, redox interactions, complexation, ion exchange, precipitation and pore adsorption are all possible mechanisms for the adsorption of heavy metal ions [77].

The modification of adsorbents in a NaOH and NaNO_3_ mixture alters the ligand environment of the adsorbent framework, thereby enhancing the adsorption properties for Cu(II). In bentonites and zeolites, a new amorphous network structure of Si–O–Si and Si–O–Al is formed, which disrupts molecular ordering and increases the adsorption capacity [24].

Adsorption at a given temperature can be quantified using mathematical equations in the form of adsorption isotherms, which relate the amount of adsorbate retained by the adsorbent (qeq_eqe) to the equilibrium concentration in solution (CeC_eCe). Two empirical models most commonly used to describe the adsorption of heavy metals at a specific temperature on various adsorbents are the Freundlich and the Langmuir isotherms. Additionally, the Temkin, Dubinin–Radushkevich, Redlich–Peterson, Koble–Corrigan and Toth isotherms are used to describe the interaction between toxic contaminants and adsorbents.

Adsorption isotherms play a key role in interpreting the mechanism of metal ion adsorption on different adsorbents. These models help elucidate the surface properties of the adsorbents and the intermolecular interactions between the adsorbed molecules and the adsorbent matrix. Isotherm and kinetic models contribute to understanding the adsorption process and depend on various factors, including the structure of the adsorbent and the physical and chemical properties of the dissolved substance.

The Langmuir model is applied to solid–liquid systems, explaining that all sites on the adsorbent surface have an equal probability of being occupied by heavy metals. In contrast, the Freundlich model characterizes a non-ideal process occurring on heterogeneous surfaces, often involving the formation of multiple adsorption layers [77].

Various kinetic models have been developed to describe the adsorption process, such as the pseudo-first-order (PFO) model, the pseudo-second-order (PSO) model, the mixed-order (MO) model, Ritchie’s equation, the Elovich model [78] and phenomenological mass-transfer models. However, there are certain issues when applying these kinetic models. The first is that the most commonly used models, PFO and PSO, are empirical and lack specific physical meaning.

From the empirical adsorption kinetic models, the widely used PFO and PSO models can be derived using Langmuir kinetics. Theoretical analysis and the literature review indicate that the PFO model represents the following conditions:

1. High initial adsorbate concentrations;
2. The initial stage of adsorption;
3. An adsorbent that contains few active sites.

In some cases, the PFO model can describe diffusion-controlled kinetics.

The PSO model, on the other hand, may represent the following conditions:

1. High initial adsorbate concentrations;
2. The final stage of adsorption;
3. An adsorbent surface rich in active sites.

In most cases, the PSO model reveals the adsorption mechanism occurring at the active sites [27].

Melichová et al. [79] utilised adsorption processes for the capture of Cu(II) using different adsorbents, including bentonite and zeolite at 20 °C, with 0.5 g of adsorbent and 100 cm^3^ of solution. By studying the adsorption of heavy metals (including Cu II), Vengris et al. [80] confirmed the suitability of using the Langmuir isotherm when using modified clays (zeolites). By studying the adsorption of Cu(II), they found the maximum capacity of the monolayer *q_m_* in the one-component system to be at the level of 83.3 and in the three-component system at the level of 80.3 mg·g^−1^. As reported in the study of Melichová et al. [79], neither positive nor negative dependence of parameters *q_m_* and n on temperature and thermal–alkaline treatment was confirmed in our case.

The Langmuir isotherms were the most relevant for fly ash. This indicates the formation of a monolayer of adsorbate on the surface of the adsorbate at all three temperatures. As the temperature increased, the nonlinear shape of the models gradually equalised to a linearly occurring dependence. For the alkaline-treated fly ash, the measured sorption data were more in accordance with the Freundlich isotherm model. Adsorption occurs on a heterogeneous surface by a multilayer mechanism. In the modified fly ash, this trend was observed at all three temperatures. By comparing the individual isotherms of fly ash and modified fly ash at particular temperatures, a positive effect of fly ash treatment is clearly observed, especially at 20 °C. At a higher temperature of 30 °C, no significant effect of thermal treatment was observed.

Isotherm models were evaluated using the statistical parameter R^2^, the coefficient of determination, which assesses the percentage of variability in the concentration of the adsorbed component at equilibrium. A higher value of the coefficient of determination correlates with a better fit of the regression model. The quality of the model is related to the chemical and mineralogical changes in the adsorbents during their thermal–alkaline treatment, which positively influences the adsorption process.

### 4.3. Evaluation of the Separation Factor

The separation factor R_L_ was calculated and evaluated from the Langmuir constant b, whose value indicates the favourability, unfavorability or linearity of the adsorption process for Cu(II) adsorption according to [67,81]. R_L_ levels, which we found in all cases, confirm the favourability of adsorption when using selected adsorbents. The stated findings are consistent with the findings of the authors presented below. In our study, according to Table 9, it was found out that the untreated original adsorbents—fly ash, bentonite and zeolite—show a decrease in the R_L_ parameter with increasing initial concentration of the sorbed medium. This fact confirms the relevance of the description of the process by the Langmuir isotherm. It quantitatively describes the formation of an adsorbate monolayer on its outer surface, where no further adsorption takes place after it is filled with Cu(II) molecules. The influence of temperature is ambiguous in the case of the original adsorbents. Alkaline-treated adsorbents exhibit more favourable values of the R_L_ parameter (close to zero) compared to their untreated forms. Such results indicate the irreversibility of the adsorption process, which was also confirmed in the evaluation by equilibrium isotherms. Treated adsorbents, especially at a temperature of 30 °C, show the chemical character of an adsorption process. Koyuncu and Kul [82] stated that the R_L_ separation factor showed that Cu(II) ions were favourably adsorbed on natural and acid-treated bentonite. Darmayanti et al. [83] determined R_L_ of Cu(II) when using fly ash at input concentrations of 10 or 20, with levels of 0.16 or 0.09 mg·dm^−3^. The favourability of Cu(II) adsorption at the indicated concentrations was also confirmed by our study.

### 4.4. Evaluation of the Models of Pseudo-First-Order and Pseudo-Second-Order Kinetics

Melichová and Ľuptáková [79] demonstrated that the values of the correlation coefficients R^2^ for a pseudo-first-order model were very low. Therefore, this kinetic model was not suitable for characterising the adsorption process. The pseudo-second-order model was more suitable for experimental data obtained during the experiments, and the correlation coefficients R^2^ obtained for the pseudo-second-order kinetical model were higher (~1). According to the study, the effect of temperature on the adsorption of copper on bentonites was minimal, due to the calculated q_e_ values, which changed only slightly with temperature change. Harja et al. [84] proved the appropriateness of the description of the adsorption mechanism of synthesised adsorbents (zeolites from ash) using pseudo-second-order kinetics. Vavouraki et al. [85] synthesised zeolites by the fusion of lignite fly ash and NaOH or KOH at 600 °C. They evaluated the effectiveness in terms of decontamination of solutions containing Cu(II) ions. The kinetic data showed that the pseudo-second-order equations described the removal process well. Copper removal was achieved mainly by the combined action of chemisorption and intraparticle diffusion. Based on a study by Joseph et al. [86], a pseudo-second-order equation was the most appropriate for describing the adsorption mechanism of Cu(II) onto fly ash derived from coal combustion. In the study, k_2_ was found to be 0.012 g·mg^−1^ min^−1^. Liu and Zhou [87] achieved a level of k_1_ of 8.9 dm^3^·min^−1^ and k_2_ of 0.0053 g·mg^−1^ min^−1^ in the adsorption of Cu(II) by bentonite. That study demonstrated a favourable description of the adsorption mechanism for pseudo-first- and pseudo-second-order models. Koyuncu and Kul [82] presented kinetic constants using natural bentonite, k_1_ 0.037 dm^3^·min^−1^ and k_2_ 0.0876 g·mg^−1^·min^−1^. Zou et al. [88] used manganese oxide-coated zeolite for Cu(II) adsorption. The k_2_ level was reported in the range of 0.625–3.29 g·mg^−1^·min^−1^. Adamczuk and Kołodyńska [89] used fly ash for the adsorption of heavy metals. They were able to better describe the adsorption of Cu(II) using the pseudo-second-order model. The constants k_2_ reached values in the range of 0.03–0.059 g·mg^−1^·min^−1^. Apiratikul and Pavasant investigated the use of modified zeolite from coal fly ash for the adsorption of heavy metals, including Cu(II) [90]. A better description of the adsorption process was confirmed for the pseudo-second-order model; the kinetics of k_2_ reached levels in the range of 0.246–0.538 kg·mol^−1^·min^−1^.

The study of adsorption kinetics provides information about the adsorption rate, the efficiency of the adsorbent used, and the mass transfer mechanisms. Knowledge of the kinetics of the process is essential for the design of adsorption systems. From the measured equilibrium data of Cu(II) concentrations, pseudo-first-order (PFO) and pseudo-second-order (PSO) kinetic models were constructed in our study (Table 11). These are the most widely used empirical models, modified by linear regression, which is the most often used method for calculating model parameters, due to its simplicity. The results show that the most suitable model was PSO, except for bentonite and zeolite at the lowest observed adsorbate initial concentration and a temperature of 10 °C, where the PFO model yielded a higher *R^2^* value. According to the study [91], the PFO model is more suitable for describing adsorption at the initial state of adsorption. This mechanism was probably dominant in the case of bentonite and zeolite in the conditions described. PFO also indicates the same rising trend of experimentally obtained values *q_e_* with the calculated *q_e_* values. At all input concentrations, which were relatively low, the dependence of the *k*_2_ parameter on temperature and adsorbent treatment is similar. According to the study [91], the PSO model represents three conditions of the process:

1. Low initial concentration of the adsorbate;
2. Description of the process in the final phase;
3. Adsorbents rich in active sites.

In general, modified materials are rich in active sites. Therefore, adsorption kinetics is dominated by adsorption at the active site. The R^2^ parameter shows a value of 1 for all modified adsorbents; for unmodified adsorbents, these values are slightly lower but still close to 1. Adsorption at the active sites of the adsorbent involves three steps:

4. External diffusion of the adsorbate through the liquid film around the solid particle of the adsorbent;
5. Internal diffusion into the pores of the adsorbent;
6. Adsorption of the adsorbate at the active sites of the adsorbent.

The difference in concentrations between the solution volume and the adsorbent surface is the driving force of external diffusion, as demonstrated by the increase in *k*_2_ with the increasing value of *c*_0_ of the adsorbate.

Kinetic models were evaluated using the statistical parameter R^2^, the coefficient of determination, which assesses the percentage of variability in the concentration of the adsorbed component at equilibrium. A higher value of the coefficient of determination correlates with a better fit of the regression model. The quality of the model is related to the chemical and mineralogical changes of the adsorbents during their thermal–alkaline treatment, which positively influences the adsorption process.

### 4.5. Evaluation of the Thermodynamic Parameters—Changes in ΔG° ΔH° and ΔS°

Thermodynamic studies provide insights into the minimum kinetic energy required for an adsorbate to bind to an adsorption site. The nature of the adsorption process (spontaneity, randomness, endothermicity or exothermicity) can be evaluated by estimating thermodynamic parameters, such as the change in Gibbs free energy (ΔG°, kJ·mol^−1^), the change in standard enthalpy (ΔH°, kJ·mol^−1^) and the change in standard entropy (ΔS°, J·mol^−1^·K^−1^).

The thermodynamic properties associated with the removal of metal ions vary due to the composition, structure and surface characteristics of the adsorbent, leading to different affinities for metal ion removal. An increased surface area and porous structure can enhance interactions and subsequent adsorption. Factors such as the presence of competing ions and shifts in pH can modify the charge distribution on both the metal ions and the adsorbent surface, thereby affecting thermodynamic equilibrium. Additionally, different metal ions exhibit distinct thermodynamic affinities due to their unique electronic configurations and charge densities [77].

The most significant difference in ∆G° values between the thermally treated and original adsorbent was observed in the case of bentonite (3–4 kJ·mol^−1^), followed by fly ash (1.5–5 kJ·mol^−1^) and finally, zeolite (2–2.5 kJ·mol^−1^). Thus, if the adsorption is a spontaneous process, ∆H°>0 points to an endothermic process. The released energy, due to thermal–alkaline treatment, was increased by 41.0 kJ·mol^−1^ in the case of fly ash. In the case of bentonite, it was increased by only 16.7 kJ·mol^−1^. Alkaline thermal treatment had the least effect on zeolite in terms of enthalpy change. Even untreated zeolite, probably due to the high porosity of the material, had a ∆*H*° value higher than its alkaline thermally treated form. Entropy is a measure of the disorder of a system. The higher the disorder is, the higher the entropy. It follows from our research that thermal–alkaline treatment of fly ash and bentonite leads to an increased value of ∆S. Zeolite shows the opposite trend, the thermal–alkaline treatment reduced the degree of the system disorder. In their study, Darmayanti et al. [92] adsorbed Cu(II) at 25 °C, 45 °C and 60 °C. The highest value of ΔG^0^ was −16.493 kJ·mol^−1^ (60 °C). In our research, the highest value of this parameter was −21.586 kJ·mol^−1^ in the case of the fly ash (30 °C) and −26.888 kJ·mol^−1^ in the case of modified fly ash (30 °C). The ∆H° value in the reference study [92] was 53.834 kJ·mol^−1^. In our study, fly ash achieved a ∆H° value of 31.09, while modified fly ash reached 72.1 kJ·mol^−1^. The highest value of ΔS^0^ in a study [92] was 211.4 J·mol^−1^·K^−1^, while in our study, the values were 173.89 (fly ash) and 324.08 (modified fly ash).

Karapinar and Donat studied the adsorption of Cu(II) and Cd(II) on natural bentonite [93]. The highest ΔG^0^ value of −29.64 kJ·mol^−1^ was observed at 60 °C. Our ΔG^0^ value was lower—for bentonite: −21.400 and for modified bentonite: −25.551 kJ·mol^−1^ (at 30 °C). In the study [93], a ∆H° level of 0.0146 kJ·mol^−1^ was achieved by adsorption. This value is much lower than that obtained in our research on bentonite (32.34 kJ·mol^−1^) and modified bentonite (49.10 kJ·mol^−1^). In the reference study [93], the ΔS^0^ value was 89.06 J·mol^−1^·K^−1^, while in our study, the values were 177.61 (bentonite) and 246.63 (modified bentonite). Panayotova studied the kinetics and thermodynamics of the removal of Cu ions from wastewater using zeolite [94]. The highest ΔG^0^ value, −4.591 kJ·mol^−1^, was achieved at 50 °C. In our study, we achieved higher ΔG^0^ values for zeolite (−16.768 kJ·mol^−1^) and modified zeolite (−18.748 kJ·mol^−1^) at 30 °C. According to the reference study, the highest ∆H° level was 27.355 kJ·mol^−1^ (32 °C). This is lower than the value obtained in our study for zeolite: 37.88 kJ·mol^−1^ (30 °C); and of modified zeolite: 29.11 kJ·mol^−1^ (30 °C). The highest level of the ΔS^0^ parameter was 99.6 J·mol^−1^·K^−1^ in the reference study, while we achieved the values of 179.28 J·mol^−1^·K^−1^ (zeolite) and 157.23 J·mol^−1^·K^−1^ (modified zeolite) at 30 °C.

### 4.6. Evaluation of Adsorption Capacity

In the study by Al-Harahsheh et al. [95], the positive effect of increasing temperature on Cu(II) adsorption was also confirmed. In this study, adsorption was carried out for 120 min using fly ash from coal combustion with an adsorbent concentration of 6.2 g·dm^−3^ (more than twice our concentration) with a temperature increase from 25.35 to 45 °C. No significant increase in adsorption capacity due to temperature was observed in the sorption of modified fly ash. This consequence was probably caused by an increase in adsorption capacity due to thermal–alkaline treatment. Thermal–alkaline treatment had a positive effect on the increase in adsorption capacity for Cu(II) sorption at all three temperatures. In the past, this finding was confirmed by a study by Chen et al. [26]. The positive effect of thermal–alkaline treatment in fly ash treatment was observed in this study [26] with the same addition of the adsorbent—2.5 g·dm^−3^ as in our research, at a temperature of 30 °C. No significant effect of temperature was observed during the adsorption of Cu(II) by bentonite at individual initial concentrations. In some cases, at the end of the sorption, which lasted for 120 min, the adsorption capacity decreased slightly to the average values. Likewise, no significant effect of temperature on adsorption by modified bentonite was observed. Li et al. studied the effect of different conditions (including temperature) on Cu(II) sorption using bentonite [96]. Farsi et al. also found an increase in Cu(II) adsorption by increasing the temperature using activated bentonite as an adsorbent [97]. In our study, a positive effect of thermal–alkaline treatment on the adsorption capacity of bentonite was observed. Thermal–alkaline treatment had a significant effect on the sorption of Cu(II) at 30 °C at an initial concentration of 18 mg·dm^−3^, when an increase in average adsorption capacity from 6.5 mg·g^−1^ to 7.2 mg·g^−1^ was observed. The increase in adsorption by thermal–alkaline treatment of bentonite was also confirmed by Abdallah [98]. In that study, the maximum percentage of removed metals was found to be more than two to three times higher compared with original bentonite, with different removal patterns for individual metals. In contrast to our study, Abdallah studied the adsorption of other PTEs (Co, Mn and Cd). This may have caused the difference in sorption between modified and unmodified bentonite in our study to be less significant. Temperature had a positive effect on the increase in adsorption capacity for the adsorption of Cu(II) by zeolite at the initial concentrations of 5, 9, 16 and 18 mg·dm^−3^. At the initial concentration of 12 mg·L^−1^, a reduction in the adsorption capacity of bentonite was observed at the temperature of 20 °C compared to the temperatures of 10 °C and 30 °C. The highest increase in adsorption capacity in the sorption of Cu(II) by zeolite was monitored at the initial concentration of 18 mg·dm^−3^, when an increase in the adsorption capacity was recorded at the levels of 5.513; 5.967 and 6.729 mg·g^−1^. Panayotova [94] investigated the removal of Cu(II) by zeolite under different conditions. The study confirmed the positive effect of increasing temperature on the sorption of Cu(II). The findings of that study are consistent with our results. No significant effect of sorption temperature on adsorption capacity was observed in the adsorption of Cu(II) by modified zeolite. A significant positive effect of thermal–alkaline treatment on the adsorption capacity of zeolite was monitored. At a temperature of 10 °C, an increase in the adsorption capacity of zeolite after thermal–alkaline treatment was observed at an initial concentration of 18 mg·dm^−3^, from 5.513 to 7.200 mg·g^−1^.

### 4.7. Evaluation of the Effect of pH on the Adsorption Process

The pH value of the aqueous solution is a significant variable that governs the adsorption of Cu(II) at the adsorbent–solution interface. The results of the study [99] revealed that the maximum adsorption of copper(II) ions onto maple sawdust reached 9.51 mg·g^−1^ at pH 6.0. The variation in the Langmuir isotherm parameters indicate the fact that the affinity of metal ions onto maple wood sawdust is pH-dependent [99]. The study investigated the adsorption behaviour of Cu(II) on Cs/PVA/PEG beads as a function of solution pH, temperature and contact time. According to the study, the maximum adsorption reached 99.99% for an initial copper ion concentration of 25 mg·dm^−3^ at pH 5, a temperature of 45 °C, a contact time of 5 h and an adsorbent dose of 1 mg·dm^−3^ [100]. Eloussaief et al. [101] studied the pH and temperature effects on the adsorption of copper ions on green and red clays from Tejera-Esghira in Medenine, Tunisia. At 20 °C, pH 5.5 and before acid-activation, the amount adsorbed by the green and red clays was 28.3 and 13.2 mg·g^−1^, respectively, and increased to 40.6 and 26.8 mg·g^−1^ after acid activation. According to the study [102], under optimal conditions (pH 7, 1 g·dm^−3^ adsorbent dose, 120 min contact time, 20 mg·dm^−3^ initial metal concentration and 20 °C), the maximum adsorption capacity of the activated bentonite was 14 ± 0.03 mg·g^−1^ for Cu^2+^, exceeding that of the natural bentonite, which had a capacity of 9.2 ± 0.04 mg·g^−1^.

Our pH measurement results fall within the alkaline range. The mine drainage itself exhibited a pH of 7.35, which, according to study [63], is at the threshold of Cu(II) precipitation into insoluble Cu(II) species. The modification of the adsorbents increased the pH, and copper was present in the form of the soluble salt Cu(OH)_2_, which had the most positive effect on adsorption onto the modified bentonite at pH 8.1–10.

## 5. Conclusions

The problem of pollution of surface streams by PTEs from mine effluents or other industrial activities can be solved by an adsorption process, using available adsorbents—zeolite, bentonite and economically and ecologically advantageous fly ash. In this study, the adsorbents zeolite, bentonite and fly ash, as well as the effect of temperature and modifications on the adsorption process, were observed. Alkaline thermal treatment of bentonite and zeolite increased the pH of the mine neutral drainage solutions, as expected due to the alkaline modification process. However, the pH of the fly ash was reduced by the modification. It was found that rising temperature increases the adsorption capacity of unmodified fly ash adsorbents, bentonite and zeolite. With modified adsorbents, the effect of temperature was not significant. However, an essential factor in increasing the adsorption capacity was the alkaline treatment of the adsorbents. The R_L_ separation factor also confirms this finding. The R_L_ values indicate the irreversibility of the adsorption process when using alkaline-treated adsorbents, which is economically advantageous, especially for waste-type materials suitable for adsorption.

The original adsorbents conformed to the Langmuir isotherm model, except for zeolite, which behaved differently and was best suited for the Temkin isotherm model. For all modified adsorbents, the Freundlich model was the most suitable one. The effect of temperature was positive for the original adsorbents, and it was the most evident in the case of fly ash. In the alkaline-treated forms of the adsorbents, the highest observed temperature of −30 °C was unfavourable for the physical course of the process. The chemical essence of the process was manifested there, which was confirmed by an increase in the value of adsorption heat from the Temkin isothermal model.

For the experimental data obtained in the experiments, a pseudo-second-order kinetic model was more suitable, describing the removal process well. Copper removal was achieved mainly by the combined action of chemisorption and intraparticle diffusion. The k_2_ levels for the modified zeolite showed a decrease with rising temperature in all cases except at the highest initial concentrations. The effect of adsorbent modification was definitely positive on the adsorption rate described by the PSO model.

Thermodynamic parameters confirm the positive effect of increasing process temperature, especially for modified adsorbents. The most energy-efficient process was observed in the case of modified fly ash and modified bentonite. Alkaline treatment has the least effect on zeolite in terms of enthalpy change. Alkaline thermal treatment of bentonite and zeolite increased the pH of the neutral mine drainage solutions, as expected from the alkaline modification process. However, the pH of the fly ash was reduced by the modification. The suitability of using adsorbents to reduce the concentration of Cu(II) in neutral mine effluents was, under the monitored conditions, as follows: at 30 °C, unmodified fly ash > modified bentonite > unmodified zeolite; at 20 and 10 °C, the same trend in the suitability of adsorbents to reduce the concentration of Cu(II) was observed—modified bentonite > modified zeolite > modified fly ash.

## Figures and Tables

**Table 1:** List of test methods for the analysis of neutral mine drainage.

Parameter /Metal	Method	
	Principle	Marking
Cu	AES-ICP	SWP 6 (ISO 11885) [41]
Mn	AES-ICP	SWP 6 (ISO 11885)
Zn	AES-ICP	SWP 6 (ISO 11885)
Fe	AES-ICP	SWP 6 (ISO 11885)
Pb	AES-ICP	SWP 6 (ISO 11885)
Cd	AES-ICP	SWP 6 (ISO 11885)
Al	AES-ICP	SWP 6 (ISO 11885)

Explanations: AES-ICP—Atomic emission spectrometry with the inductively coupled plasma.

**Table 2:** List of test methods for the analysis of fly ash.

Parameter /Metal	Method	
	Principle	Marking
dry matter	gravimetrically	STN ISO 11465 < (SWP 2) [42]
pH/H_2_O	potentiometrically	ISO 10390 (SWP 8) [43]
S	EA-TCD	SWP 4 (ISO 15178) [44]
Al	AES-ICP	SWP 6 (ISO 11885)
B	AES-ICP	SWP 6 (ISO 11885)
Cr	AES-ICP	SWP 6 (ISO 11885)
Mn	AES-ICP	SWP 6 (ISO 11885)
Cu	AES-ICP	SWP 6 (ISO 11885)
Na	AES-ICP	SWP 6 (ISO 11885)
Zn	AES-ICP	SWP 6 (ISO 11885)
Fe	AES-ICP	SWP 6 (ISO 11885)
Cd	AES-ICP	SWP 6 (ISO 11885)
Pb	AES-ICP	SWP 6 (ISO 11885)
Ni	AES-ICP	SWP 6 (ISO 11885)
Hg	AAS AMA	SWP 15 (CSI) 75 7440 l [45]
Mg	AES-ICP	SWP 6 (ISO 11885). SWP 19 Mehlich III
Ca	AES-ICP	SWP 6 (ISO 11885). SWP 19 Mchlich III
K	AES-ICP	SWP 6 (ISO 11885). SWP 19 Mehlich III
P	AES-ICP	SWP 6 (ISO 11885). SWP 19 Mehlich III
Cu	AES-ICP	SWP 6 (ISO 11885). SWP 19 Mehlich III
Fe	AES-ICP	SWP 6 (ISO 11885). SWP 19 Mehlich III
Mn	AES-ICP	SWP 6 (ISO 11885). SWP 19 Mehlich III
Zn	AES-ICP	SWP 6 (ISO 11885). SWP 19 Mehlich III

Explanations: EA-TCD—elemental analysis with thermal conductivity detection; AES-ICP—Atomic emission spectrometry with the inductively coupled plasma; SWP—standard work procedure; AAS-AMA—atomic absorption spectrometry–advanced mercury analyser.

**Table 3:** Normalised results of quantitative X-ray powder diffraction analysis (weight %).

Adsorbent Sample	Fly Ash, Before Treatment	Fly Ash, After Treatment	Bentonite, Before Treatment	Bentonite, After Treatment	Zeolite, Before Treatment	Zeolite, After Treatment
Method	Rie	Rie	RJ	Rie	RJ	RJ
Mineral	[wt.%]					
quartz	22	24	6	3	-	-
K-feldspar	-	7	11	5	<1	3
plagioclase	5	35	-	-	4	11
biotite	-	-	2	2	<1	3
clinoptilolite/heulandite	-	-	-	-	76	29
opal-CT	-	-	6	-	15	-
arcanite	17	-	-	-	-	-
cancrinite	-	-	-	16	-	-
calcite	29	34	-	<1	-	4
portlandite	16	-	-	-	-	-
hydroxylapatite	5	-	-	-	-	-
periclase	6	-	-	-	-	-
kaolinite	-	-	2	-	-	-
smectite	-	-	73	1	3	<1
amorphous content	-	-	-	72	-	50
Sum	100	100	100	100	100	100

RJ—RockJock [61]; Rie—Rietveld—[61].

**Table 4:** Concentrations of metal elements in the NMD sample and their physicochemical characteristics.

Metal Content [µg·dm^−3^]	Ag	Ba	Cd	Co	As
*LV*	5		0.08 for the 1st hardness class up to 0.25 5th hardness class	50	7.5
MV	<0.05	12.66	21.43	11.14	1
**Metal Content [µg·dm^−3^]**	**Pb**	**Se**	**Sb**	**Sn**	**Sr**
*LV*	7.2	20			
MV	1.7	1.3	0.61	0.39	1814.39
**Metal Content [µg·dm^−3^]**	Cu *	**Fe**	**Zn**	**Tl**	**Al**
*LV*	1.1 for the 1st and 2nd hardness class and 8.8 for 4th and 5th hardness class	total Fe 2 mg·dm^−3^	7.8 for the 1st and 2nd hardness class and 52 for 4th and 5th hardness class		200
MV	17.2	<10	4821.6	0.48	141
pH [-]	7.6				

* Cu exceeded the limit values; LV—limit value according to the Regulation of the Government of the Slovak Republic No. 269/2010 Coll. MV—measured value.

**Table 5:** Chemical composition and elemental analysis of bentonite and zeolite obtained from the supplier of a specific bearing.

Chemical Composition [%]									
**Adsorbent**	SiO_2_	Al_2_O_3_	Fe_2_O_3_	CaO	MgO	TiO_2_	Na_2_O	K_2_O	MnO
**Bentonite**	60.18–73.8	11.56–24.90	2.15–3.39	1.23–2.17	1.09–3.29	0.11–0.20	0.29–0.91	0.42–1.18	0.02–0.06
**Zeolite**	64.18–75.50	10.93–14.80	0.12–2.45	1.43–11.68	0.29–1.43	-	0.10–2.97	1.24–4.24	–
**Elemental Analysis [** **mg·kg^−1^]**									
**Adsorbent**	Copper	Antimony	Zinc	Iron	Lead	Cadmium	Arsenic	Aluminium	
**Bentonite**	7.40	23.4	64.4	23.711	12.4	-	6.3	121.198	
**Zeolite**	3.12	0.15	-	8.044	8.39	0.045	0.91	66.685	

**Table 6:** Characteristics of fly ash.

Parameter					
Dry matter	pH/H_2_O	S	Al	B	Cr
%	-	mg·kg^−1^	mg·kg^−1^	mg·kg^−1^	mg·kg^−1^
99.97	12.11	19,400	17,448	295	14.5
Mn	Cu	Na	Zn	Fe	Cd
mg·kg^−1^	mg·kg^−1^	mg·kg^−1^	mg·kg^−1^	mg·kg^−1^	mg·kg^−1^
4771	117	2099	1649	11,253	11.8
Pb	Ni	Hg	Mg	Ca	K
mg·kg^−1^	mg·kg^−1^	μg·kg^−1^	mg·kg^−1^	mg·kg^−1^	mg·kg^−1^
43.0	15.6	3.04	0.561	62,419	88,632

**Table 7:** BET characteristics of the sorbents used [m^2^·g^−1^].

Adsorbent	BET Surface Area (m^2^·g^−1^)
fly ash	15.433
bentonite	43.098
zeolite	33.506
modified fly ash	16.449
modified bentonite	27.472
modified zeolite	10.098

**Table 8:** Langmuir, Freundlich and Temkin constants for the adsorption of Cu(II) on adsorbents at different temperatures—10, 20 and 30 °C.

Adsorbent	Temperature	Freundlich Constants		Langmuir Constants		Temkin Constants	
	t [°C]	Kf [mg·g^−1^]	n	q_m_ [mg·g^−1^]	b [dm^3^·mg^−1^]	A [dm^3^·g^−1^]	b_t_ [J·mol^−1^]
Fly ash	10	4.630	2.091	7.819	1.611	17.546	1451.230
	20	6.582	3.688	6.549	9.828	103.571	1743.130
	30	19.059	1.134	36.467	0.735	21.089	694.579
Modified fly ash	10	9.368	2.057	8.484	5.783	72.422	1401.750
	20	10.127	1.934	10.087	4.236	46.214	1140.310
	30	8.162	4.564	7.685	15.949	18 778	3975.660
Bentonite	10	5.061	4.766	5.937	5.831	136.082	2350.720
	20	5.530	2.810	7.222	3.184	24.269	1390.920
	30	5.512	3.199	7.541	3.567	31.875	1523.130
Modified bentonite	10	14.233	1.447	15.962	2.142	23.974	771.812
	20	18.681	1.798	23.083	2.923	130.58	1150.080
	30	7.732	4.970	34.109	1.319	9483.3	3289.896
Zeolite	10	2.991	2.083	8.839	0.482	2.951	998.073
	20	2.096	1.482	8.540	0.478	2.651	1152.540
	30	5.665	1.316	22.162	0.347	10.314	1020.170
Modified zeolite	10	16.530	1.595	12.588	4.520	29.748	708.488
	20	9.447	1.299	19.405	0.812	13.139	806.982
	30	8.024	2.194	9.610	3.311	25.902	1046.620

**Table 9:** RL values calculated from the Langmuir constants.

Temperature (°C)	Adsorbent c_0_ [mg·dm^−3^]														
	Fly ash					Bentonite					Zeolite				
	5	9	12	16	18	5	9	12	16	18	5	9	12	16	18
10	0.083	0.048	0.037	0.028	0.025	0.086	0.049	0.038	0.028	0.025	0.429	0.294	0.238	0.190	0.173
20	0.052	0.030	0.023	0.017	0.015	0.053	0.030	0.023	0.017	0.015	0.379	0.253	0.202	0.160	0.145
30	0.303	0.195	0.153	0.120	0.108	0.106	0.062	0.047	0.036	0.032	0.205	0.125	0.097	0.075	0.067
	Modified fly ash					Modified bentonite					Modified zeolite				
	5	9	12	16	18	5	9	12	16	18	5	9	12	16	18
10	0.024	0.014	0.010	0.008	0.007	0.118	0.069	0.053	0.040	0.036	0.003	0.002	0.001	0.001	0.001
20	0.040	0.022	0.017	0.013	0.011	6.18 × 10^−4^	3.44 × 10^−4^	2.58 × 10^−4^	1.93 × 10^−4^	1.72 × 10^−4^	1.80 × 10^−1^	1.09 × 10^−1^	8.37 × 10^−2^	6.41 × 10^−2^	5.74 × 10^−2^
30	0.005	0.003	0.002	0.001	0.001	*	*	*	*	*	0.105	0.061	0.047	0.035	0.032

* Absolute removal of Cu(II) from the solution was achieved after the first half an hour of adsorption. Therefore, it was not possible to determine the RL.

**Table 10:** Statistical data of Langmuir, Freundlich and Temkin constants for the adsorption of Cu(II) on adsorbents at different temperatures—10, 20 and 30 °C.

Adsorbent	Isotherm	F-Test	*p*-Level *
Bentonite 10 °C	Freundlich	89.46	0.000
	Langmuir	91.09	0.000
	Temkin	89.91	0.000
Bentonite 20 °C	Freundlich	107.39	0.000
	Langmuir	112.48	0.000
	Temkin	110.30	0.000
Bentonite 30 °C	Freundlich	276.68	0.000
	Langmuir	389.02	0.000
	Temkin	335.48	0.000
Zeolite 10 °C	Freundlich	207.76	0.000
	Langmuir	242.03	0.000
	Temkin	250.50	0.000
Zeolite 20 °C	Freundlich	175.21	0.000
	Langmuir	180.63	0.000
	Temkin	184.42	0.000
Zeolite 30 °C	Freundlich	1439.30	0.000
	Langmuir	1217.71	0.000
	Temkin	693.33	0.000
Fly ash 10 °C	Freundlich	311.94	0.000
	Langmuir	318.06	0.000
	Temkin	309.29	0.000
Fly ash 20 °C	Freundlich	107.23	0.000
	Langmuir	113.41	0.000
	Temkin	109.95	0.000
Fly ash 30 °C	Freundlich	1220.39	0.000
	Langmuir	1261.66	0.000
	Temkin	1087.93	0.000
Modified bentonite 10 °C	Freundlich	469.78	0.000
	Langmuir	485.05	0.000
	Temkin	494.01	0.000
Modified bentonite 20 °C	Freundlich	19.04	0.000
	Langmuir	500.27	0.000
	Temkin	610.23	0.000
Modified bentonite 30 °C	Freundlich	92.31	0.000
	Langmuir	408.56	0.000
	Temkin	320.79	0.000
Modified zeolite 10 °C	Freundlich	126.54	0.000
	Langmuir	90.40	0.000
	Temkin	70.45	0.000
Modified zeolite 20 °C	Freundlich	1777.37	0.000
	Langmuir	1597.5	0.000
	Temkin	1371.74	0.000
Modified zeolite 30 °C	Freundlich	167.97	0.000
	Langmuir	167.56	0.000
	Temkin	169.88	0.000
Modified fly ash 10 °C	Freundlich	387.57	0.000
	Langmuir	379.54	0.000
	Temkin	365.31	0.000
Modified fly ash 20 °C	Freundlich	1328.07	0.000
	Langmuir	1550.38	0.000
	Temkin	1243.12	0.000
Modified fly ash 30 °C	Freundlich	286.93	0.000
	Langmuir	185.54	0.000
	Temkin	357.03	0.000

* All *p*-level values were below 0.000.

**Table 11:** Pseudo-first-order and pseudo-second-order models.

Adsorbent	Temperature	q_e_ Experim. [mg·g^−1^]	Pseudo-First-Order				Pseudo-Second-Order			
	[°C]		k_1_ [dm^3^·min^−1^]	q_e_ [mg·g^−1^]	R^2^	Equation	k_2_ [g·mg^−1^ min^−1^]	q_e_ [mg·g^−1^]	R^2^	Equation
5 [mg·dm^−3^]										
Fly ash	10	1.934	0.0109	1.745	0.579	y = 0.0109x + 0.5565	0.903	0.389	0.998	y = 2.5728x + 7.3327
	20	1.951	0.0108	1.830	0.534	y = 0.0108x + 0.6045	2.579	0.393	1	y = 2.546x + 2.513
	30	1.968	0.0108	1.837	0.532	y = 0.0108x + 0.6084	2.408	0.396	1	y = 2.5264x + 2.6508
Modified fly ash	10	1.984	0.0107	1.876	0.512	y = 0.0107x + 0.6289	6.069	0.397	1	y = 2.5191x + 1.0457
	20	1.981	0.0107	1.879	0.509	y = 0.0107x + 0.6309	7.774	0.396	1	y = 2.5243x + 0.8197
	30	2.000	0.0107	1.899	0.502	y = 0.0107x + 0.6413	24.300	0.400	1	y = 2.4995x + 0.2571
Bentonite	10	1.860	0.0133	1.214	0.886	y = 0.0133x + 0.1939	0.089	0.439	0.824	y = 2.2801x + 58.422
	20	1.871	0.0111	1.653	0.634	y = 0.0111x + 0.5027	0.569	0.384	0.994	y = 2.6014x + 11.893
	30	1.832	0.0106	1.755	0.559	y = 0.0106x + 0.5625	1.707	0.390	1	y = 2.5664x + 3.8582
Modified bentonite	10	1.967	0.0106	1.881	0.505	y = 0.0106x + 0.632	13.184	0.394	1	y = 2.5412x + 0.4898
	20	2.000	0.6295	1.877	0.516	y = 0.0108x + 0.6295	5.342	0.161	1	y = 2.4949x + 1.1651
	30	2.000	0.0108	1.894	0.506	y = 0.0108x + 0.6385	15.777	0.401	1	y = 2.4967x + 0.3951
Zeolite	10	1.578	0.0108	1.182	0.922	y = 0.0108x + 0.1674	0.140	0.330	0.839	y = 3.032x + 65.646
	20	1.656	0.0096	1.484	0.673	y = 0.0096x + 0.3948	0.416	0.316	0.953	y = 3.1679x + 24.097
	30	1.926	0.0115	1.518	0.727	y = 0.0115x + 0.4177	0.284	0.390	0.974	y = 2.5667x + 23.22
Modified zeolite	10	1.979	0.0107	1.880	0.5068	y = 0.0107x + 0.6315	8.161	0.395	1	y = 2.5314x + 0.7852
	20	1.942	0.0106	1.866	0.509	y = 0.0106x + 0.6236	8.394	0.388	1	y = 2.5741x + 0.7894
	30	1.938	0.0108	1.806	0.5441	y = 0.0108x + 0.5911	1.676	0.390	1	y = 2.5627x + 3.9187
9 [mg·dm^−3^]										
Fly ash	10	3.331	0.0145	2.186	0.552	y = 0.0145x + 0.7821	1.043	0.373	0.998	y = 2.6843x + 6.9117
	20	3.476	0.0145	2.351	0.509	y = 0.0145x + 0.8549	7.848	0.387	1	y = 2.5811x + 0.8489
	30	3.513	0.0147	2.297	0.531	y = 0.0147x + 0.8315	1.845	0.393	0.999	y = 2.5437x + 3.5069
Modified fly ash	10	3.530	0.0145	2.377	0.504	y = 0.0145x + 0.866	14.673	0.392	1	y = 2.5498x + 0.4431
	20	3.539	0.0145	2.380	0.503	y = 0.0145x + 0.8673	14.325	0.393	1	y = 2.544x + 0.4518
	30	3.562	0.0146	2.390	0.502	y = 0.0146x + 0.8715	17.820	0.396	1	y = 2.5258x + 0.358
Bentonite	10	3.244	0.0146	1.879	0.659	y = 0.0146x + 0.6305	0.127	0.298	0.961	y = 2.8046x + 26.378
	20	3.228	0.0147	2.073	0.593	y = 0.0147x + 0.7291	0.663	0.368	0.995	y = 2.7159x + 11.127
	30	3.478	0.0152	2.130	0.592	y = 0.0152x + 0.7561	3.092	0.385	1	y = 2.5943x + 2.177
Modified bentonite	10	3.518	0.0145	2.381	0.502	y = 0.0145x + 0.8675	37.302	0.391	1	y = 2.5579x + 0.1754
	20	3.600	0.0148	2.336	0.524	y = 0.0148x + 0.8484	5.322	0.161	1	y = 2.49x + 2.5051
	30	3.600	0.0147	2.399	0.503	y = 0.0147x + 0.8751	21.811	0.400	1	y = 2.4976x + 0.286
Zeolite	10	3.113	0.0163	1.485	0.849	y = 0.0163x + 0.3957	0.101	0.389	0.841	y = 2.5689x + 65.018
	20	3.002	0.0145	1.929	0.635	y = 0.0145x + 0.657	0.463	0.348	0.987	y = 2.8755x + 17.859
	30	3.376	0.0146	2.100	0.583	y = 0.0146x + 0.7418	0.596	0.373	0.991	y = 2.68x + 12.044
Modified zeolite	10	3.599	0.0147	2.385	0.507	y = 0.0147x + 0.869	7.085	0.400	1	y = 2.4989x + 0.8814
	20	3.510	0.0146	2.323	0.519	y = 0.0146x + 0.8429	2.472	0.390	1	y = 2.5611x + 2.6529
	30	3.478	0.0145	2.325	0.515	y = 0.0145x + 0.8438	3.140	0.386	1	y = 2.5929x + 2.1411
12 [mg·dm^−3^]										
Fly ash	10	4.181	0.0184	1.750	0.772	y = 0.0184x + 0.5597	0.117	0.400	0.865	y = 2.5027x + 53.539
	20	4.616	0.0166	2.545	0.531	y = 0.0166x + 0.9342	5.728	0.401	1	y = 2.4952x + 1.0869
	30	4.707	0.0169	2.520	0.545	y = 0.0169x + 0.9242	1.161	0.397	0.999	y = 2.5212x + 5.4732
Modified fly ash	10	4.635	0.0163	2.675	0.497	y = 0.0163x + 0.9839	14.858	0.386	1	y = 2.5906x − 0.4517
	20	4.716	0.0164	2.675	0.501	y = 0.0164x + 0.984	5.728	0.401	1	y = 2.4952x + 1.0869
	30	4.726	0.0165	2.664	0.504	y = 0.0165x + 0.9798	7.841	0.393	1	y = 2.5438x + 0.8253
Bentonite	10	4.597	0.0177	2.191	0.643	y = 0.0177x + 0.7844	0.303	0.405	0.979	y = 2.4698x + 20.162
	20	4.674	0.0179	2.150	0.661	y = 0.0179x + 0.7654	5.728	0.401	1	y = 2.4952x + 1.0869
	30	4.200	0.0162	2.394	0.553	y = 0.0162x + 0.8731	5.527	0.391	1	y = 2.5566x + 1.1827
Modified bentonite	10	4.743	0.0165	2.671	0.504	y = 0.0165x + 0.9823	9.691	0.395	1	y = 2.5311x + 0.6611
	20	4.800	0.0166	2.652	0.511	y = 0.0166x + 0.9752	5.728	0.401	1	y = 2.4952x + 1.0869
	30	4.800	0.0166	2.672	0.508	y = 0.0166x + 0.983	5.728	0.401	1	y = 2.4952x + 1.0869
Zeolite	10	4.111	0.0164	2.268	0.588	y = 0.0164x + 0.8189	0.682	0.352	0.994	y = 2.8409x + 11.84
	20	3.585	0.0153	2.050	0.621	y = 0.0153x + 0.7176	5.728	0.401	1	y = 2.4952x + 1.0869
	30	4.453	0.0166	2.411	0.561	y = 0.0166x + 0.8801	0.879	0.375	0.997	y = 2.6643x + 8.0793
Modified zeolite	10	4.721	0.0166	2.659	0.509	y = 0.0166x + 0.9778	8.540	0.396	1	y = 2.5251x + 0.7466
	20	4.613	0.0164	2.616	0.511	y = 0.0164x + 0.9615	5.728	0.401	1	y = 2.4952x + 1.0869
	30	4.685	0.0165	2.642	0.509	y = 0.0165x + 0.9717	5.542	0.391	1	y = 2.5559x + 1.1788
16 [mg·dm^−3^]										
Fly ash	10	5.898	0.0192	2.355	0.642	y = 0.0192x + 0.8564	0.253	0.383	0.964	y = 2.6128x + 27.027
	20	6.205	0.0187	2.800	0.542	y = 0.0187x + 1.0295	0.944	0.392	0.997	y = 2.5529x + 6.9036
	30	6.302	0.0186	2.901	0.523	y = 0.0186x + 1.0651	1.795	0.396	0.999	y = 2.5253x + 3.5534
Modified fly ash	10	6.255	0.0183	2.982	0.504	y = 0.0183x + 1.0925	9.881	0.390	1	y = 2.5639x + 0.6653
	20	6.210	0.0182	3.011	0.497	y = 0.0182x + 1.1023	10.971	0.387	1	y = 2.581x − 0.6072
	30	6.296	0.0184	3.005	0.501	y = 0.0184x + 1.1002	29.117	0.394	1	y = 2.5407x + 0.2217
Bentonite	10	5.991	0.0189	2.569	0.591	y = 0.0189x + 0.9437	0.438	0.386	0.988	y = 2.5883x + 15.311
	20	6.105	0.0194	2.412	0.635	y = 0.0194x + 0.8804	0.249	0.400	0.959	y = 2.4998x + 25.106
	30	6.235	0.0187	2.861	0.532	y = 0.0187x + 1.0512	3.247	0.394	1	y = 2.5391x + 1.9858
Modified bentonite	10	6.299	0.0185	2.980	0.507	y = 0.0185x + 1.092	7.320	0.395	1	y = 2.5344x + 0.8775
	20	6.382	0.0185	2.988	0.508	y = 0.0185x + 1.0945	4.831	0.400	1	y = 2.5015x + 1.2952
	30	6.400	0.0186	2.988	0.509	y = 0.0186x + 1.0946	4.973	0.401	1	y = 2.4921x + 1.2489
Zeolite	10	5.703	0.0187	2.242	0.659	y = 0.0187x + 0.8073	0.220	0.361	0.937	y = 2.7723x + 34.893
	20	5.704	0.0189	2.268	0.655	y = 0.0189x + 0.8187	0.218	0.369	0.930	y = 2.7077x + 33.705
	30	6.000	0.0184	2.785	0.536	y = 0.0184x + 1.0244	1.127	0.377	0.998	y = 2.65x + 6.2315
Modified zeolite	10	6.374	0.0185	3.004	0.505	y = 0.0185x + 1.1001	9.829	0.399	1	y = 2.5081x + 0.64
	20	6.164	0.0183	2.953	0.507	y = 0.0183x + 1.0829	7.039	0.386	1	y = 2.589x + 0.9523
	30	6.319	0.0185	2.956	0.511	y = 0.0185x + 1.084	3.270	0.394	1	y = 2.5386x + 1.9709
18 [mg·dm^−3^]										
Fly ash	10	6.654	0.0193	2.624	0.589	y = 0.0193x + 0.9647	0.391	0.367	0.978	y = 2.7266x + 19.033
	20	7.123	0.0196	3.000	0.533	y = 0.0196x + 1.0985	1.361	0.399	0.999	y = 2.5055x + 4.6108
	30	7.067	0.0192	3.104	0.509	y = 0.0192x + 1.1328	4.218	0.393	1	y = 2.5451x + 1.5356
Modified fly ash	10	7.055	0.0191	3.149	0.500	y = 0.0191x + 1.147	63.965	0.391	1	y = 2.5543x + 0.102
	20	7.045	0.0191	3.151	0.499	y = 0.0191x + 1.1478	54.778	0.391	1	y = 2.5585x + 0.1195
	30	7.144	0.0193	3.127	0.507	y = 0.0193x + 1.1401	5.627	0.397	1	y = 2.5182x + 1.127
Bentonite	10	7.026	0.0202	2.613	0.614	y = 0.0202x + 0.9605	0.285	0.407	0.970	y = 2.4587x + 21.188
	20	6.897	0.0207	2.411	0.664	y = 0.0207x + 0.8799	0.193	0.411	0.951	y = 2.4345x + 30.643
	30	6.482	0.0192	2.823	0.552	y = 0.0192x + 1.0378	29.836	0.384	1	y = 2.6019x + 0.2269
Modified bentonite	10	7.051	0.0195	3.004	0.530	y = 0.0195x + 1.1001	1.560	0.395	1	y = 2.5321x + 4.1095
	20	7.200	0.0194	3.106	0.513	y = 0.0194x + 1.1334	3.157	0.160	1	y = 2.4964x + 1.9742
	30	7.200	0.0193	3.162	0.503	y = 0.0193x + 1.1513	13.032	0.400	1	y = 2.4979x + 0.4788
Zeolite	10	5.513	0.019	2.273	0.657	y = 0.019x + 0.8212	0.297	0.325	0.969	y = 3.079x + 31.961
	20	5.967	0.0186	2.556	0.586	y = 0.0186x + 0.9383	0.467	0.334	0.982	y = 2.9962x + 19.214
	30	6.729	0.0189	2.983	0.518	y = 0.0189x + 1.093	1.870	0.371	0.998	y = 2.6971x + 3.8898
Modified zeolite	10	7.200	0.0193	3.160	0.503	y = 0.0193x + 1.1507	11.797	0.400	1	y = 2.4976x + 0.5288
	20	6.955	0.0192	3.087	0.5096	y = 0.0192x + 1.1272	5.063	0.388	1	y = 2.5794x + 1.3141
	30	6.918	0.019	3.119	0.5017	y = 0.019x + 1.1375	27.124	0.385	1	y = 2.6004x + 0.2493

**Table 12:** Values of the thermodynamic parameters ΔG^0^, ΔH^0^ and ΔS^0^.

Adsorbent	Temperature	ΔG^0^	ΔH^0^	ΔS
	[°C]	[kJ·mol^−1^]	[kJ·mol^−1^]	[J·mol^−1^·K^−1^]
Fly ash	10	−18.113	31.09	173.89
	20	−19.969		
	30	−21.586		
Modified fly ash	10	−20.312	72.10	324.08
	20	−21.522		
	30	−26.888		
Bentonite	10	−17.861	32.34	177.61
	20	−19.924		
	30	−21.400		
Modified bentonite	10	−20.634	49.10	246.63
	20	−23.434		
	30	−25.551		
Zeolite	10	−13.144	37.88	179.28
	20	−14.119		
	30	−16.768		
Modified zeolite	10	−15.578	29.11	157.23
	20	−16.614		
	30	−18.748		

**Table 13:** Descriptive statistics of adsorption capacity.

Adsorbent	Time [min]	Valid N	Mean [mg·g^−1^]	Confidence	Confidence	Std. Dev.
Fly ash	30	6	1.67	1.64	1.70	0.03
Fly ash	30	6	2.93	2.92	2.95	0.02
Fly ash	30	6	1.77	1.74	1.79	0.02
Fly ash	30	6	3.92	3.84	3.99	0.07
Fly ash	30	6	5.00	4.93	5.07	0.06
Fly ash	60	6	1.79	1.79	1.80	0.01
Fly ash	60	6	3.09	3.07	3.11	0.02
Fly ash	60	6	3.52	3.43	3.60	0.08
Fly ash	60	6	4.45	4.44	4.47	0.02
Fly ash	60	6	5.30	5.28	5.32	0.02
Fly ash	90	6	1.87	1.86	1.87	0.01
Fly ash	90	6	3.26	3.25	3.26	0.01
Fly ash	90	6	4.07	4.04	4.09	0.02
Fly ash	90	6	5.64	5.61	5.67	0.03
Fly ash	90	6	5.85	5.84	5.87	0.01
Fly ash	120	6	1.93	1.93	1.94	0.01
Fly ash	120	6	3.33	3.32	3.35	0.01
Fly ash	120	6	4.18	4.17	4.19	0.01
Fly ash	120	6	5.90	5.89	5.90	0.01
Fly ash	120	6	6.65	6.64	6.67	0.02
Bentonite	30	6	0.74	0.70	0.78	0.04
Bentonite	30	6	2.25	2.22	2.27	0.02
Bentonite	30	6	2.99	2.96	3.02	0.03
Bentonite	30	6	4.58	4.55	4.60	0.03
Bentonite	30	6	5.01	4.87	5.15	0.13
Bentonite	60	6	1.44	1.42	1.46	0.02
Bentonite	60	6	2.41	2.38	2.43	0.03
Bentonite	60	6	4.16	4.14	4.18	0.02
Bentonite	60	6	5.13	5.10	5.15	0.02
Bentonite	60	6	5.46	5.42	5.51	0.04
Bentonite	90	6	1.86	1.85	1.87	0.01
Bentonite	90	6	2.74	2.71	2.77	0.03
Bentonite	90	6	4.60	4.58	4.61	0.01
Bentonite	90	6	5.99	5.97	6.01	0.02
Bentonite	90	6	7.03	7.02	7.03	0.01
Bentonite	120	6	1.86	1.85	1.87	0.01
Bentonite	120	6	3.24	3.16	3.32	0.08
Bentonite	120	6	4.60	4.58	4.61	0.01
Bentonite	120	6	5.99	5.97	6.01	0.02
Bentonite	120	6	7.03	7.02	7.03	0.01
Zeolite	30	6	0.81	0.80	0.83	0.01
Zeolite	30	6	1.34	1.32	1.36	0.02
Zeolite	30	6	3.15	3.12	3.18	0.03
Zeolite	30	6	3.66	3.63	3.69	0.03
Zeolite	30	6	3.42	3.39	3.44	0.03
Zeolite	60	6	0.89	0.85	0.92	0.03
Zeolite	60	6	2.01	1.98	2.03	0.02
Zeolite	60	6	3.98	3.96	4.01	0.03
Zeolite	60	6	3.86	3.73	3.99	0.12
Zeolite	60	6	4.58	4.54	4.63	0.04
Zeolite	90	6	1.35	1.32	1.38	0.03
Zeolite	90	6	2.91	2.90	2.92	0.01
Zeolite	90	6	4.06	4.03	4.08	0.02
Zeolite	90	6	4.97	4.93	5.01	0.04
Zeolite	90	6	5.43	5.37	5.50	0.06
Zeolite	120	6	1.58	1.56	1.59	0.02
Zeolite	120	6	3.11	3.07	3.15	0.04
Zeolite	120	6	4.11	4.11	4.12	0.00
Zeolite	120	6	5.70	5.66	5.75	0.04
Zeolite	120	6	5.51	5.47	5.56	0.04
Modified fly ash	30	6	1.94	1.93	1.94	0.01
Modified fly ash	30	6	3.49	3.49	3.50	0.01
Modified fly ash	30	6	4.66	4.65	4.66	0.00
Modified fly ash	30	6	6.16	6.16	6.16	0.00
Modified fly ash	30	6	7.04	7.03	7.06	0.01
Modified fly ash	60	6	1.96	1.96	1.97	0.00
Modified fly ash	60	6	3.52	3.51	3.52	0.00
Modified fly ash	60	6	4.68	4.67	4.69	0.01
Modified fly ash	60	6	6.26	6.20	6.32	0.05
Modified fly ash	60	6	7.07	7.03	7.11	0.04
Modified fly ash	90	6	1.97	1.97	1.97	0.00
Modified fly ash	90	6	3.52	3.51	3.52	0.00
Modified fly ash	90	6	4.63	4.63	4.64	0.00
Modified fly ash	90	6	6.18	6.17	6.18	0.01
Modified fly ash	90	6	7.03	7.02	7.03	0.01
Modified fly ash	120	6	1.98	1.98	1.99	0.00
Modified fly ash	120	6	3.53	3.53	3.53	0.00
Modified fly ash	120	6	4.64	4.63	4.64	0.01
Modified fly ash	120	6	6.25	6.25	6.26	0.00
Modified fly ash	120	6	7.05	7.05	7.06	0.00
Modified bentonite	30	6	1.95	1.95	1.95	0.00
Modified bentonite	30	6	3.50	3.49	3.51	0.01
Modified bentonite	30	6	4.68	4.67	4.69	0.01
Modified bentonite	30	6	6.14	6.13	6.16	0.01
Modified bentonite	30	6	6.31	6.28	6.34	0.03
Modified bentonite	60	6	1.96	1.95	1.96	0.00
Modified bentonite	60	6	3.52	3.51	3.52	0.00
Modified bentonite	60	6	4.70	4.70	4.71	0.00
Modified bentonite	60	6	6.28	6.27	6.28	0.00
Modified bentonite	60	6	6.93	6.90	6.96	0.03
Modified bentonite	90	6	1.96	1.96	1.96	0.00
Modified bentonite	90	6	3.51	3.51	3.51	0.00
Modified bentonite	90	6	4.72	4.71	4.72	0.00
Modified bentonite	90	6	6.29	6.29	6.30	0.00
Modified bentonite	90	6	6.97	6.96	6.97	0.01
Modified bentonite	120	6	1.97	1.97	1.97	0.00
Modified bentonite	120	6	3.52	3.52	3.52	0.00
Modified bentonite	120	6	4.74	4.74	4.75	0.00
Modified bentonite	120	6	6.30	6.30	6.30	0.00
Modified bentonite	120	6	7.05	7.04	7.06	0.01
Modified zeolite	30	6	1.95	1.95	1.95	0.00
Modified zeolite	30	6	3.53	3.53	3.53	0.00
Modified zeolite	30	6	4.61	4.61	4.62	0.01
Modified zeolite	30	6	6.28	6.28	6.28	0.00
Modified zeolite	30	6	7.13	7.13	7.13	0.00
Modified zeolite	60	6	1.96	1.96	1.96	0.00
Modified zeolite	60	6	3.56	3.56	3.57	0.00
Modified zeolite	60	6	4.72	4.72	4.72	0.00
Modified zeolite	60	6	6.34	6.33	6.34	0.00
Modified zeolite	60	6	7.14	7.14	7.15	0.00
Modified zeolite	90	6	1.96	1.95	1.96	0.00
Modified zeolite	90	6	3.58	3.58	3.59	0.00
Modified zeolite	90	6	4.79	4.79	4.79	0.00
Modified zeolite	90	6	6.36	6.36	6.36	0.00
Modified zeolite	90	6	7.17	7.12	7.22	0.05
Modified zeolite	120	6	1.98	1.98	1.98	0.00
Modified zeolite	120	6	3.60	3.60	3.60	0.00
Modified zeolite	120	6	4.72	4.58	4.86	0.13
Modified zeolite	120	6	6.37	6.37	6.38	0.01
Modified zeolite	120	6	7.17	7.12	7.22	0.05

## Data Availability

The original contributions presented in this study are included in the article/Supplementary Materials; further inquiries can be directed to the corresponding author.

## Supplementary Materials

The following supporting information can be downloaded at https://www.mdpi.com/article/10.3390/ma18194552/s1, Isotherms of adsorbents in relation to temperature before and after treatment are presented in Figures S1–S27.

## References

[1] Pereira L.B., Vicentini R., Ottoboni L.M.M. (2014). Changes in the Bacterial Community of Soil from a Neutral Mine Drainage Channel. PLoS ONE.

[2] Cheng K.Y., Acuña C.R., Kaksonen A.H., Esslemont G., Douglas G.B. (2024). Sequential Hydrotalcite Precipitation, Microbial Sulfate Reduction and In Situ Hydrogen Sulfide Removal for Neutral Mine Drainage Treatment. Sci. Total Environ..

[3] Jarvis A.P., Gandy C.J., Webb J.A. (2023). Controls on the Generation and Geochemistry of Neutral Mine Drainage: Evidence from Force Crag Mine, Cumbria, UK. Minerals.

[4] Opitz J., Timms W., Drebenstedt C., Paul M. (2016). Mine Water Discharge Quality—A Review of Classification Frameworks. Mining Meets Water—Conflicts and Solutions.

[5] Marmier V., Plante B., Demers I., Benzaazoua M. (2025). Neutral Mine Drainage Prediction for Different Waste Rock Lithologies—Case Study of Canadian Malartic. J. Geochem. Explor..

[6] Ding Y., Shen S.Z., Sun H., Sun K., Liu F. (2014). Synthesis of L-Glutathione-Capped-ZnSe Quantum Dots for the Sensitive and Selective Determination of Copper Ion in Aqueous Solutions. Sens. Actuators B Chem..

[7] Akar S.T., Akar T., Kaynak Z., Anilan B., Cabuk A., Tabak Ö., Demir T.A., Gedikbey T. (2009). Removal of Copper(II) Ions from Synthetic Solution and Real Wastewater by the Combined Action of Dried *Trametes versicolor* Cells and Montmorillonite. Hydrometallurgy.

[8] Prepilková V., Poništ J., Belčáková I., Ďuricová A., Salva J., Schwarz M., Samešová D. (2024). Comparison of Copper and Zinc Sorption Depending on Temperature and Sorbents. Pol. J. Environ. Stud..

[9] Samešová D., Pochyba A., Ďuricová A., Poništ J., Prepilková V.Š., Schwarz M., Veverková D., Salva J., Schmidtová J. (2025). Comparative Adsorption of Cu(II), Zn(II), Cd(II), and Mn(II) from Aquatic Solution and Neutral Mine Drainage Using Paper Sludge. Water.

[10] Ryu S., Naidu G., Hasan Johir M.A., Choi Y., Jeong S., Vigneswaran S. (2019). Acid Mine Drainage Treatment by Integrated Submerged Membrane Distillation–Sorption System. Chemosphere.

[11] Olegario-Sanchez E., Pelicano C.M. (2017). Characterization of Philippine Natural Zeolite and Its Application for Heavy Metal Removal from Acid Mine Drainage (AMD). Key Eng. Mater..

[12] Feng G., Ma J., Zhang X., Zhang Q., Xiao Y., Ma Q., Wang S. (2019). Magnetic Natural Composite Fe_3_O_4_-Chitosan@bentonite for Removal of Heavy Metals from Acid Mine Drainage. J. Colloid Interface Sci..

[13] Wulandari E., Hidayat A.E., Moersidik S.S. (2020). Comparison of Copper Adsorption Effectivity in Acid Mine Drainage Using Natural Zeolite and Synthesized Zeolite. IOP Conf. Ser. Earth Environ. Sci..

[14] Mohammed N.H., Atta M., Yaacub W.Z.W. (2017). Remediation of Heavy Metals by Using Industrial Waste by Products in Acid Mine Drainage. Am. J. Eng. Appl. Sci..

[15] Zhang T., Tu Z., Lu G., Duan X., Yi X., Guo C., Dang Z. (2017). Removal of Heavy Metals from Acid Mine Drainage Using Chicken Eggshells in Column Mode. J. Environ. Manag..

[16] Fu F., Wang Q. (2011). Removal of Heavy Metal Ions from Wastewaters: A Review. J. Environ. Manag..

[17] Jiang F., Yin S., Zhang L., Peng J., Ju S., Miller J.D., Wang X. (2018). Solvent Extraction of Cu(II) from Sulfate Solutions Containing Zn(II) and Fe(III) Using an Interdigital Micromixer. Hydrometallurgy.

[18] Edebali S., Pehlivan E. (2016). Evaluation of Chelate and Cation Exchange Resins to Remove Copper Ions. Powder Technol..

[19] Wang J., Chen C. (2009). Biosorbents for Heavy Metals Removal and Their Future. Biotechnol. Adv..

[20] Gupta V.K., Nayak A., Agarwal S. (2015). Bioadsorbents for Remediation of Heavy Metals: Current Status and Their Future Prospects. Environ. Eng. Res..

[21] Ince M., İnce O.K. (2017). An Overview of Adsorption Technique for Heavy Metal Removal from Water/Wastewater: A Critical Review. Int. J. Pure Appl. Sci..

[22] Lucaci A.R., Bulgariu D., Popescu M.-C., Bulgariu L. (2020). Adsorption of Cu(II) Ions on Adsorbent Materials Obtained from Marine Red Algae *Callithamnion corymbosum* Sp.. Water.

[23] Savastru E., Bulgariu D., Zamfir C.-I., Bulgariu L. (2022). Application of *Saccharomyces cerevisiae* in the Biosorption of Co(II), Zn(II) and Cu(II) Ions from Aqueous Media. Water.

[24] Zhao X., Zhao H., Huang X., Wang L., Liu F., Hu X., Li J., Zhang G., Ji P. (2021). Effect and Mechanisms of Synthesis Conditions on the Cadmium Adsorption Capacity of Modified Fly Ash. Ecotoxicol. Environ. Saf..

[25] Huang X., Zhao H., Zhang G., Li J., Yang Y., Ji P. (2020). Potential of Removing Cd(II) and Pb(II) from Contaminated Water Using a Newly Modified Fly Ash. Chemosphere.

[26] He K., Chen Y., Tang Z., Hu Y. (2016). Removal of Heavy Metal Ions from Aqueous Solution by Zeolite Synthesized from Fly Ash. Environ. Sci. Pollut. Res..

[27] Król M. (2019). Hydrothermal Synthesis of Zeolite Aggregate with Potential Use as a Sorbent of Heavy Metal Cations. J. Mol. Struct..

[28] Naiya T.K., Bhattacharya A.K., Mandal S., Das S.K. (2009). The Sorption of Lead(II) Ions on Rice Husk Ash. J. Hazard. Mater..

[29] Rate A.W. (2010). Sorption of Cadmium(II) and Copper(II) by Soil Humic Acids: Temperature Effects and Sorption Heterogeneity. Chem. Ecol..

[30] Le A.D.T., Samri D., Rahim M., Douzane O., Promis G., Langlet T. (2015). Effect of Temperature-Dependent Sorption Characteristics on the Hygrothermal Behavior of Hemp Concrete. Energy Procedia.

[31] Aston J.E., Apel W.A., Lee B.D., Peyton B.M. (2010). Effects of Cell Condition, pH, and Temperature on Lead, Zinc, and Copper Sorption to *Acidithiobacillus caldus* Strain BC13. J. Hazard. Mater..

[32] Xiao B., Dai Q., Yu X., Yu P., Zhai S., Liu R., Guo X., Liu J., Chen H. (2018). Effects of Sludge Thermal-Alkaline Pre-treatment on Cationic Red X-GRL Adsorption onto Pyrolysis Biochar of Sewage Sludge. J. Hazard. Mater..

[33] Choi C.L., Park M., Lee D.H., Kim J.-E., Park B.-Y., Choi J. (2001). Salt-Thermal Zeolitization of Fly Ash. Environ. Sci. Technol..

[34] Querol X., Moreno N., Umaña J.C., Alastuey A., Hernández E., López-Soler A., Plana F. (2002). Synthesis of Zeolites from Coal Fly Ash: An Overview. Int. J. Coal Geol..

[35] Števulová N., Junák J. (2014). Alkalicky aktivované spojivo na báze popolčeka. Chem. Listy.

[36] Singh A.K., Masto R.E., Hazra B., Esterle J., Singh P.K., Singh A.K., Masto R.E., Hazra B., Esterle J., Singh P.K. (2020). Genesis and Characteristics of Coal and Biomass Ash. Ash from Coal and Biomass Combustion.

[37] Lexa J., Štohl J., Konečný V. (1999). The Banská Štiavnica Ore District: Relationship between Metallogenetic Processes and the Geological Evolution of a Stratovolcano. Miner. Depos..

[38] Števko M., Sejkora J., Malíková R. (2018). New Data on Supergene Minerals from the Banská Štiavnica Deposit (Slovak Republic). Bull. Mineral. Petrol..

[39] (2023). Kvalita Vody. Odber Vzoriek. Časť 1: Pokyny na Návrhy Programov Odberu Vzoriek a Techniky Odberu Vzoriek.

[40] (2007). Kvalita Vody. Odber Vzoriek na Mikrobiologickú Analýzu.

[41] (2007). Water Quality—Determination of Selected Elements by Inductively Coupled Plasma Optical Emission Spectrometry (ICP-OES).

[42] (2001). Kvalita Pôdy. Stanovenie Obsahu Sušiny a Hmotnostného Obsahu Vody. Gravimetrická Metóda.

[43] (2022). Zemina, Upravené Bioodpady a Kaly. Stanovenie PH.

[44] (2000). Soil Quality—Determination of Total Sulfur by Dry Combustion.

[45] eStudio.cz ČSN 75 7440 (757440). https://www.technicke-normy-csn.cz/csn-75-7440-757440-226378.html.

[46] Skousen J., Jacobs J.A. (2014). Stream Characterization for Acid Mine Drainage. Acid Mine Drainage, Rock Drainage, and Acid Sulfate Soils.

[47] Hillier S. (1999). Use of an Air Brush to Spray Dry Samples for X-Ray Powder Diffraction. Clay Miner..

[48] Yumpu.com DIFFRAC.EVA Tutorial. https://www.yumpu.com/en/document/view/34435692/diffraceva-tutorial.

[49] DIFFRAC.TOPAS. https://www.bruker.com/en/products-and-solutions/diffractometers-and-x-ray-microscopes/x-ray-diffractometers/diffrac-suite-software/diffrac-topas.html.

[50] Cheary R.W., Coelho A. (1992). A Fundamental Parameters Approach to X-Ray Line-Profile Fitting. J. Appl. Crystallogr..

[51] McGinnety J.A. (1972). Redetermination of the Structures of Potassium Sulphate and Potassium Chromate: The Effect of Electrostatic Crystal Forces upon Observed Bond Lengths. Acta Crystallogr. B.

[52] Hassan I., Grundy H.D. (1991). The Crystal Structure of Basic Cancrinite, Ideally Na_8_Al_6_Si_6_O_242_·3H_2_O. Can. Mineral..

[53] Comodi P., Fumagalli P., Montagnoli M., Zanazzi P.F. (2004). A Single-Crystal Study on the Pressure Behavior of Phlogopite and Petrological Implications. Am. Mineral..

[54] Hughes J.M., Cameron M., Crowley K.D. (1989). Structural Variations in Natural F, OH, and Cl Apatites. Am. Mineral..

[55] Angel R.J., Ross N.L., Zhao J., Sochalski-Kolbus L., Krüger H., Schmidt B.C. (2013). Structural Controls on the Anisotropy of Tetrahedral Frameworks: The Example of Monoclinic Feldspars. Eur. J. Mineral..

[56] Hazen R. (1976). Effects of Temperature and Pressure on the Cell Dimension and X-Ray Temperature Factors of Periclase. Am. Mineral..

[57] Gualtieri A.F. (2000). Accuracy of XRPD QPA Using the Combined Rietveld–RIR Method. J. Appl. Cryst..

[58] Desgranges L., Grebille D., Calvarin G., Chevrier G., Floquet N., Niepce J.-C. (1993). Hydrogen Thermal Motion in Calcium Hydroxide: Ca(OH)_2_. Acta Crystallogr. B.

[59] Antao S.M., Hassan I., Wang J., Lee P.L., Toby B.H. (2008). State-of-the-Art High-Resolution Powder X-Ray Diffraction (HRPXRD) Illustrated with Rietveld Structure Refinement of Quartz, Sodalite, Tremolite, and Meionite. Can. Mineral..

[60] Gournis D., Lappas A., Karakassides M.A., Többens D., Moukarika A. (2008). A Neutron Diffraction Study of Alkali Cation Migration in Montmorillonites. Phys. Chem. Miner..

[61] Eberl D.D. (2003). User Guide to RockJock—A Program for Determining Quantitative Mineralogy from X-Ray Diffraction Data.

[62] Środoń J., Drits V.A., McCarty D.K., Hsieh J.C.C., Eberl D.D. (2001). Quantitative X-Ray Diffraction Analysis of Clay-Bearing Rocks from Random Preparations. Clays Clay Miner..

[63] Šuránek M., Melichová Z., Mirković M.M., Ivanović M., Pavlović V.B., Kljajević L., Nenadović S. (2023). The Study of Cu(II) Adsorption onto Synthetically Modified Geopolymers. Sustainability.

[64] Duxson P., Fernández-Jiménez A., Provis J.L., Lukey G.C., Palomo A., van Deventer J.S.J. (2007). Geopolymer Technology: The Current State of the Art. J. Mater. Sci..

[65] Sari A., Tuzen M., Citak D., Soylak M. (2007). Equilibrium, Kinetic and Thermodynamic Studies of Adsorption of Pb(II) from Aqueous Solution onto Turkish Kaolinite Clay. J. Hazard. Mater..

[66] Han R., Zhang J., Zou W., Shi J., Liu H. (2005). Equilibrium Biosorption Isotherm for Lead Ion on Chaff. J. Hazard. Mater..

[67] Melichová Z., Ľuptáková A. (2016). Removing Lead from Aqueous Solutions Using Different Low-Cost Abundant Adsorbents. Desal. Water Treat..

[68] Obaid S.A. (2020). Langmuir, Freundlich and Temkin Adsorption Isotherms and Kinetics for the Removal of *Artichoke Tournefortii* Straw from Agricultural Waste. J. Phys. Conf. Ser..

[69] Sampranpiboon P. (2014). Equilibrium Isotherm Models for Adsorption of Zinc(II) Ion from Aqueous Solution on Pulp Waste. Wseas Trans. Environ. Dev..

[70] Edet U.A., Ifelebuegu A.O. (2020). Kinetics, Isotherms, and Thermodynamic Modeling of the Adsorption of Phosphates from Model Wastewater Using Recycled Brick Waste. Processes.

[71] Javadian H., Ghorbani F., Tayebi H., Asl S.H. (2015). Study of the Adsorption of Cd(II) from Aqueous Solution Using Zeolite-Based Geopolymer, Synthesized from Coal Fly Ash; Kinetic, Isotherm and Thermodynamic Studies. Arab. J. Chem..

[72] Lagergren S. (1907). Zur Theorie der sogenannten Adsorption gelöster Stoffe. Z. Phys. Chem. Kolloid..

[73] Ho Y.S., McKay G. (1999). Pseudo-Second Order Model for Sorption Processes. Process Biochem..

[74] Walpole R.E. (2016). Probability & Statistics for Engineers & Scientists.

[75] El-Enein S.A., Okbah M.A., Hussain S.G., Soliman N.F., Ghounam H.H. (2020). Adsorption of Selected Metals Ions in Solution Using Nano-Bentonite Particles: Isotherms and Kinetics. Environ. Process..

[76] Vitek R., Masini J.C. (2023). Nonlinear Regression for Treating Adsorption Isotherm Data to Characterize New Sorbents: Advantages over Linearization Demonstrated with Simulated and Experimental Data. Heliyon.

[77] Raji Z., Karim A., Karam A., Khalloufi S. (2023). Adsorption of Heavy Metals: Mechanisms, Kinetics, and Applications of Various Adsorbents in Wastewater Remediation—A Review. Waste.

[78] Elovich S.Y., Larinov O.G. (1962). Theory of Adsorption from Solutions of Non Electrolytes on Solid (I) Equation Adsorption from Solutions and the Analysis of Its Simplest Form, (II) Verification of the Equation of Adsorption Isotherm from Solutions. Izv. Akad. Nauk SSSR Otd. Khimicheskikh Nauk.

[79] Melichová Z., Ďuricová A., Samešová D., Nagyová I. (2017). Hodnotenie Rizík Vybraných Kovových Prvkov vo Vodách.

[80] Vengris T., Binkienė R., Sveikauskaitė A. (2001). Nickel, Copper and Zinc Removal from Waste Water by a Modified Clay Sorbent. Appl. Clay Sci..

[81] Melichová Z., Handzušová M. (2016). Removal of Cu(II) Ions from Aqueous Solutions by Adsorption onto Natural Bentonites. Solid State Phenom..

[82] Koyuncu H., Kul A.R. (2014). An Investigation of Cu(II) Adsorption by Native and Activated Bentonite: Kinetic, Equilibrium and Thermodynamic Study. J. Environ. Chem. Eng..

[83] Darmayanti L., Notodarmodjo S., Damanhuri E., Mukti R.R. (2018). Removal of Copper(II) Ions in Aqueous Solutions by Sorption onto Alkali Activated Fly Ash. MATEC Web Conf..

[84] Harja M., Buema G., Sutiman D.-M., Munteanu C., Bucur D. (2012). Low Cost Adsorbents Obtained from Ash for Copper Removal. Korean J. Chem. Eng..

[85] Vavouraki A., Bartzas G., Komnitsas K. (2020). Synthesis of Zeolites from Greek Fly Ash and Assessment of Their Copper Removal Capacity. Minerals.

[86] Joseph I.V., Tosheva L., Doyle A.M. (2020). Simultaneous Removal of Cd(II), Co(II), Cu(II), Pb(II), and Zn(II) Ions from Aqueous Solutions via Adsorption on FAU-Type Zeolites Prepared from Coal Fly Ash. J. Environ. Chem. Eng..

[87] Liu Z., Zhou S. (2010). Adsorption of Copper and Nickel on Na-Bentonite. Process Saf. Environ. Prot..

[88] Zou W., Han R., Chen Z., Jinghua Z., Shi J. (2006). Kinetic Study of Adsorption of Cu(II) and Pb(II) from Aqueous Solutions Using Manganese Oxide Coated Zeolite in Batch Mode. Colloids Surf. A Physicochem. Eng. Asp..

[89] Adamczuk A., Kołodyńska D. (2015). Equilibrium, Thermodynamic and Kinetic Studies on Removal of Chromium, Copper, Zinc and Arsenic from Aqueous Solutions onto Fly Ash Coated by Chitosan. Chem. Eng. J..

[90] Apiratikul R., Pavasant P. (2008). Sorption of Cu^2+^, Cd^2+^, and Pb^2+^ Using Modified Zeolite from Coal Fly Ash. Chem. Eng. J..

[91] Wang J., Guo X. (2020). Adsorption Kinetic Models: Physical Meanings, Applications, and Solving Methods. J. Hazard. Mater..

[92] Darmayanti L., Notodarmodjo S., Damanhuri E. (2017). Removal of Copper(II) Ions in Aqueous Solutions by Sorption onto Fly Ash. J. Eng. Technol. Sci..

[93] Karapinar N., Donat R. (2009). Adsorption Behaviour of Cu^2+^ and Cd^2+^ onto Natural Bentonite. Desalination.

[94] Panayotova M.I. (2001). Kinetics and Thermodynamics of Copper Ions Removal from Wastewater by Use of Zeolite. Waste Manag..

[95] Al-Harahsheh M.S., Al Zboon K., Al-Makhadmeh L., Hararah M., Mahasneh M. (2015). Fly Ash Based Geopolymer for Heavy Metal Removal: A Case Study on Copper Removal. J. Environ. Chem. Eng..

[96] Li J., Hu J., Sheng G., Zhao G., Huang Q. (2009). Effect of pH, Ionic Strength, Foreign Ions and Temperature on the Adsorption of Cu(II) from Aqueous Solution to GMZ Bentonite. Colloids Surf. A Physicochem. Eng. Asp..

[97] Farsi A., Javid N., Malakootian M. (2019). Investigation of Adsorption Efficiency of Cu^2+^ and Zn^2+^ by Red Soil and Activated Bentonite from Acid Copper Mine Drainage. Desal. Water Treat..

[98] Abdallah S.M. (2010). Towards a Safer Environment: Zeolitization of Bentonite and Its Potentialities for Removing Heavy Metals from Wastewater. J. Soil Sci. Agric. Eng..

[99] Rahman M.S., Islam M.R. (2009). Effects of pH on Isotherms Modeling for Cu(II) Ions Adsorption Using Maple Wood Sawdust. Chem. Eng. J..

[100] Trikkaliotis D.G., Christoforidis A.K., Mitropoulos A.C., Kyzas G.Z. (2020). Adsorption of Copper Ions onto Chitosan/Poly(Vinyl Alcohol) Beads Functionalized with Poly(Ethylene Glycol). Carbohydr. Polym..

[101] Eloussaief M., Jarraya I., Benzina M. (2009). Adsorption of Copper Ions on Two Clays from Tunisia: pH and Temperature Effects. Appl. Clay Sci..

[102] Eleraky M.I., Razek T.M.A., Hasani I.W., Fahim Y.A. (2025). Adsorptive Removal of Lead, Copper, and Nickel Using Natural and Activated Egyptian Calcium Bentonite Clay. Sci. Rep..

