# Supplementary material for: Comparison of Cu(II) Adsorption Using Fly Ash and Natural Sorbents During Temperature Change and Thermal–Alkaline Treatment
Source: Materials (Basel). 2025 Sep 30;18(19):4552. doi: 10.3390/ma18194552 (PMC12525864; doi:10.3390/ma18194552)
Supplement: Supplementary file 1 [file materials-18-04552-s001.zip › materials-3841341-supplementary.pdf]

Isotherms of adsorbents in relation to temperature before and after treatment are presented in Figures S1–S27.

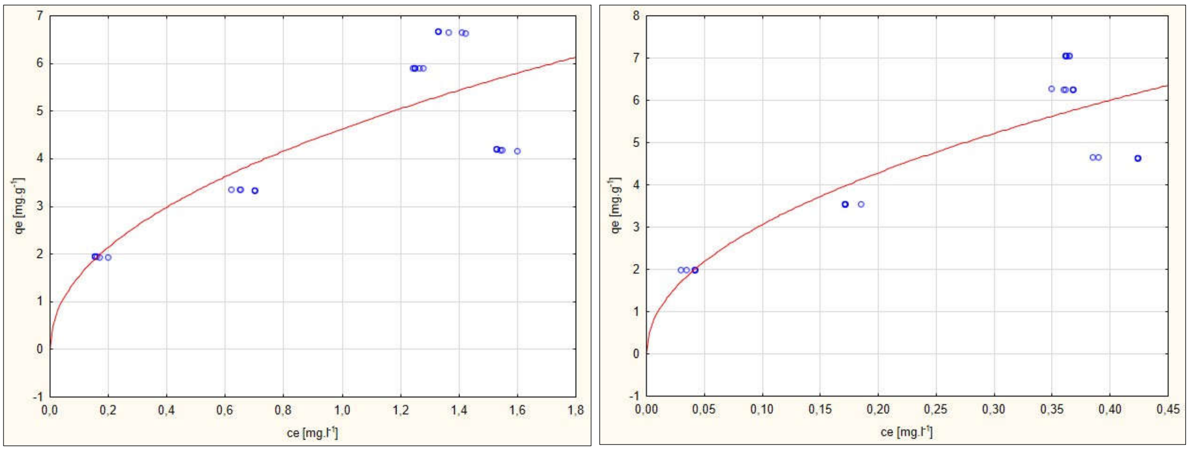

Figure S1. Nonlinear correlations of Cu(II) on fly ash before treatment (left) and after treatment (right) at 10 °C, using the Freundlich isotherm model.

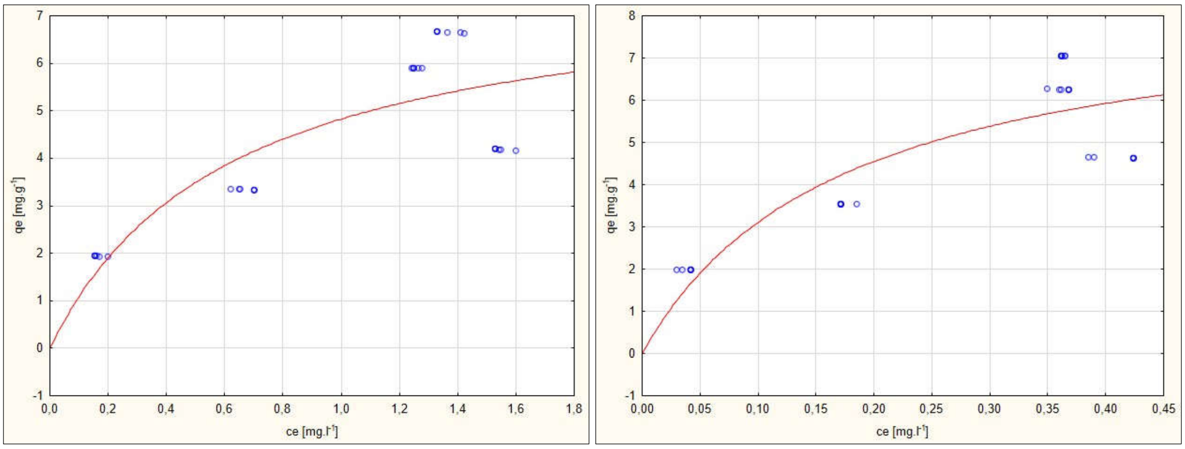

Figure S2. Nonlinear correlations of Cu(II) on fly ash before treatment (left) and after treatment (right) at 10 °C, using the Langmuir isotherm model.

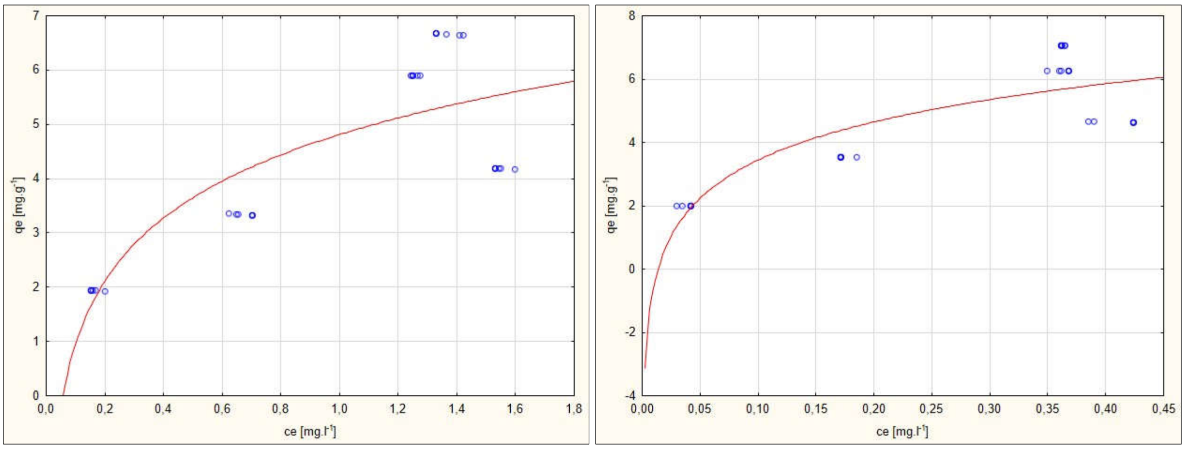

Figure S3. Nonlinear correlations of Cu(II) on fly ash before treatment (left) and after treatment (right) at 10 °C, using the Temkin isotherm model.

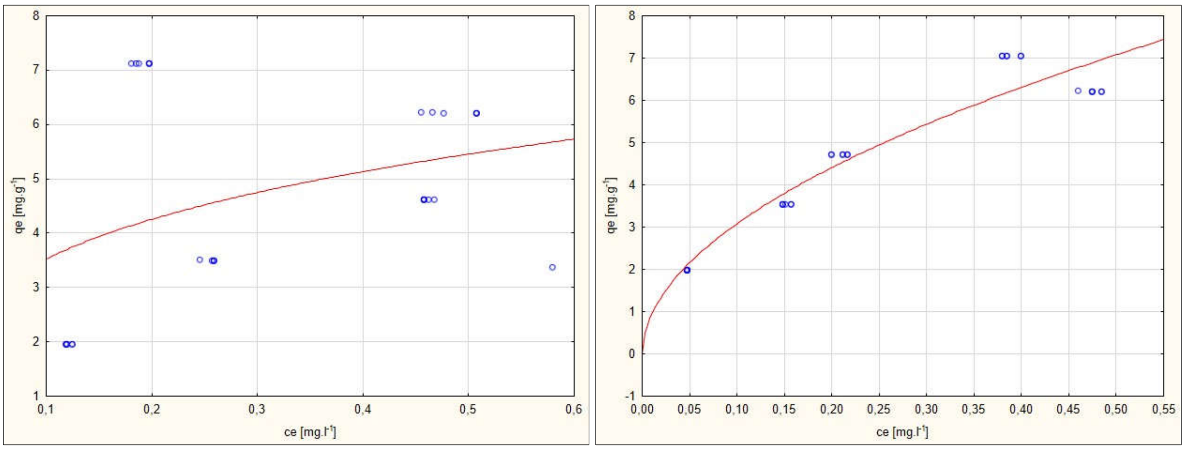

Figure S4. Nonlinear correlations of Cu(II) on fly ash before treatment (left) and after treatment (right) at 20 °C, using the Freundlich isotherm model.

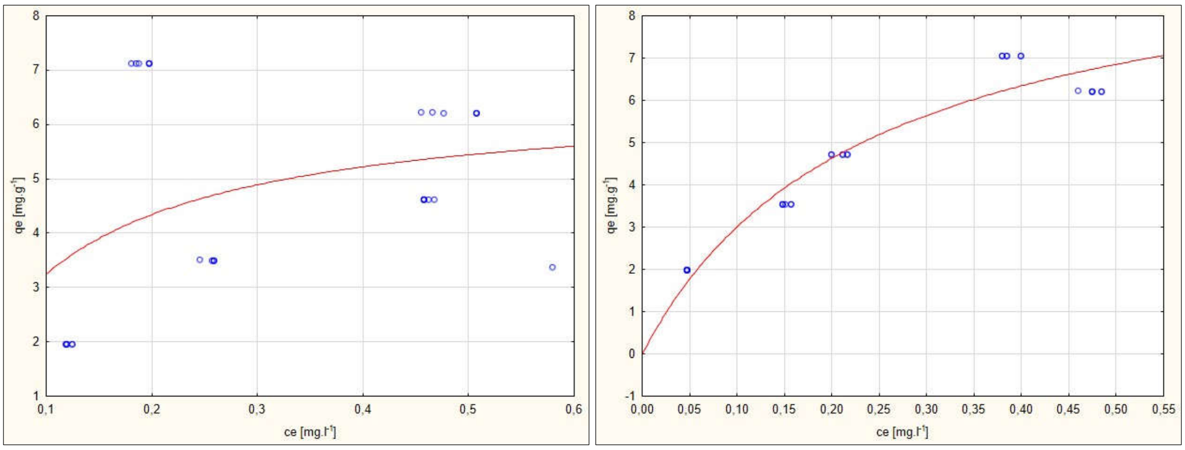

Figure S5. Nonlinear correlations of Cu(II) on fly ash before treatment (left) and after treatment (right) at 20 °C, using the Langmuir isotherm model.

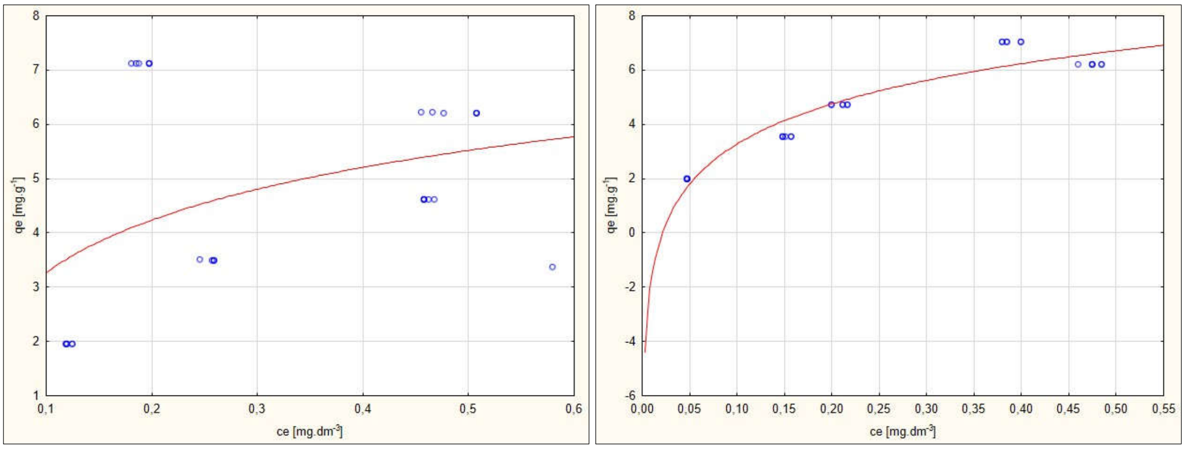

Figure S6. Nonlinear correlations of Cu(II) on fly ash before treatment (left) and after treatment (right) at 20 °C, using the Temkin isotherm model.

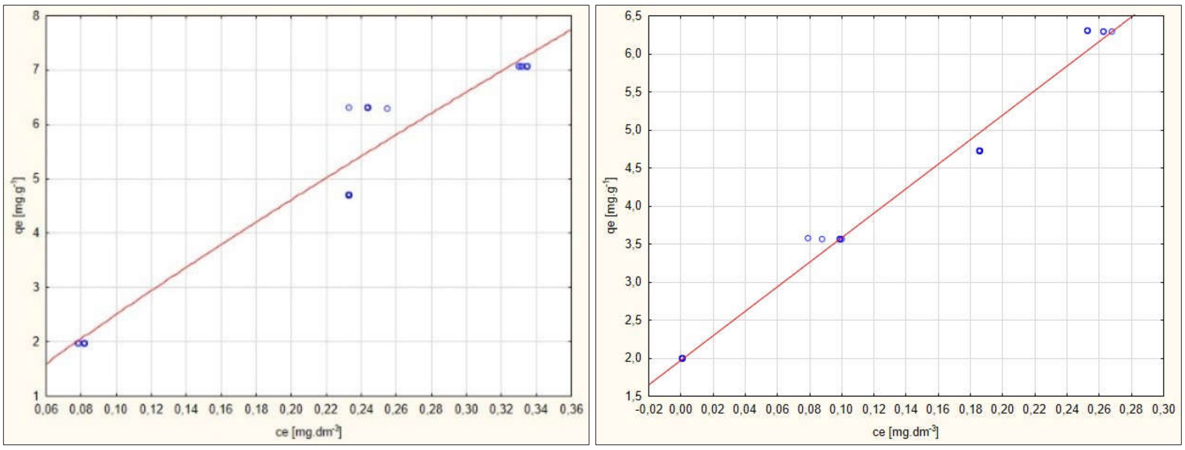

Figure S7. Nonlinear correlations of Cu(II) on fly ash before treatment (left) and after treatment (right) at 30 °C, using the Freundlich isotherm model.

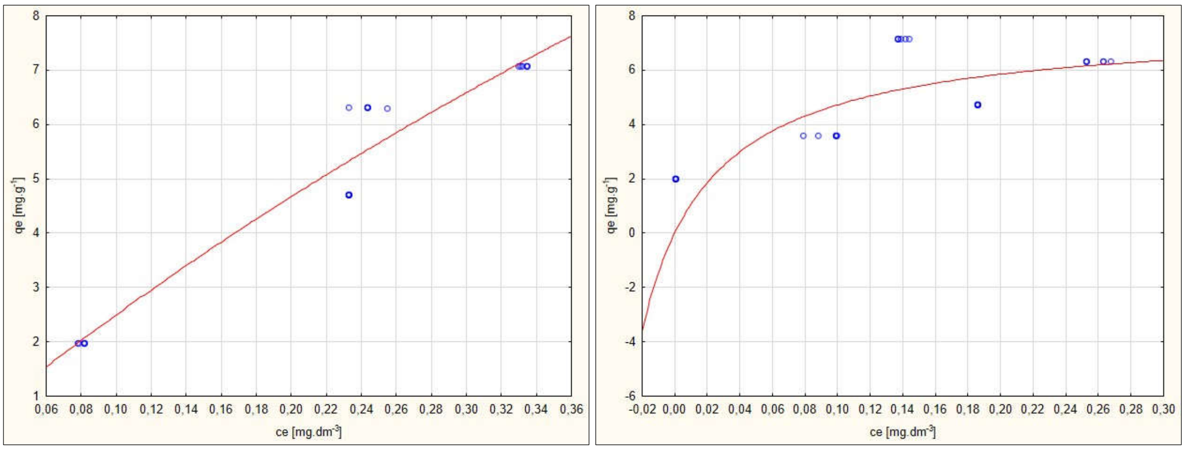

Figure S8. Nonlinear correlations of Cu(II) on fly ash before treatment (left) and after treatment (right) at 30 °C, using the Langmuir isotherm model.

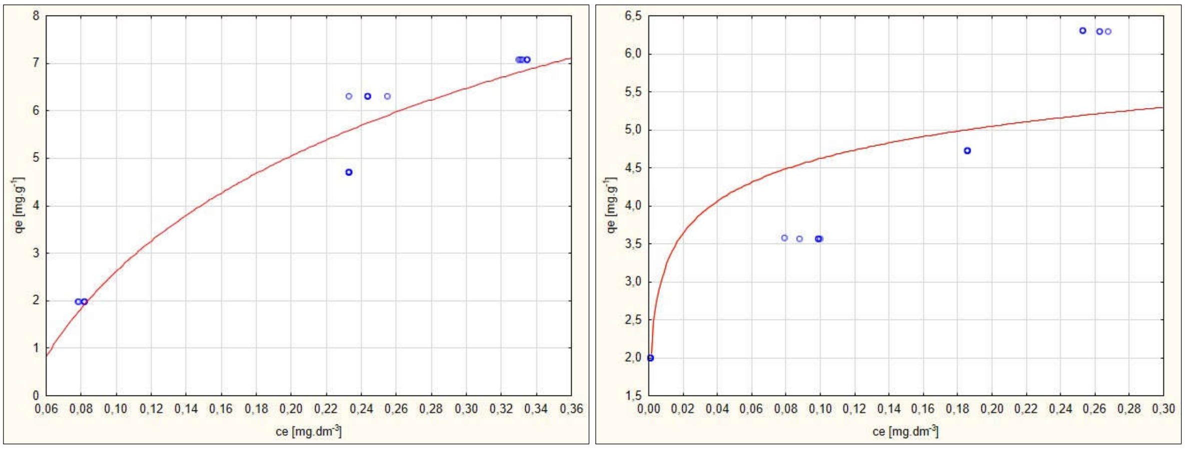

Figure S9. Nonlinear correlations of Cu(II) on fly ash before treatment (left) and after treatment (right) at 30 °C, using the Temkin isotherm model.

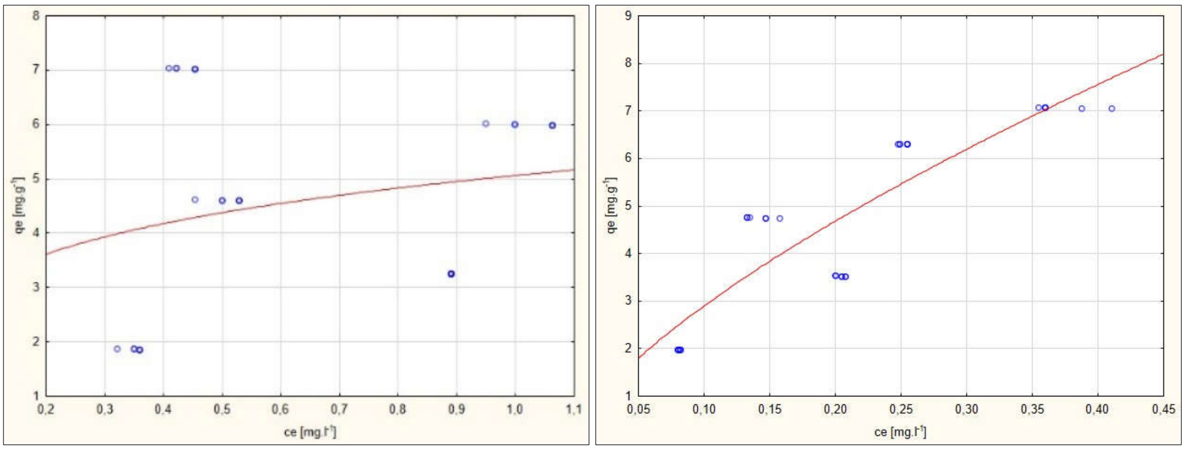

Figure S10. Nonlinear correlations of Cu(II) on bentonite before treatment (left) and after treatment (right) at 10 °C, using the Freundlich isotherm model.

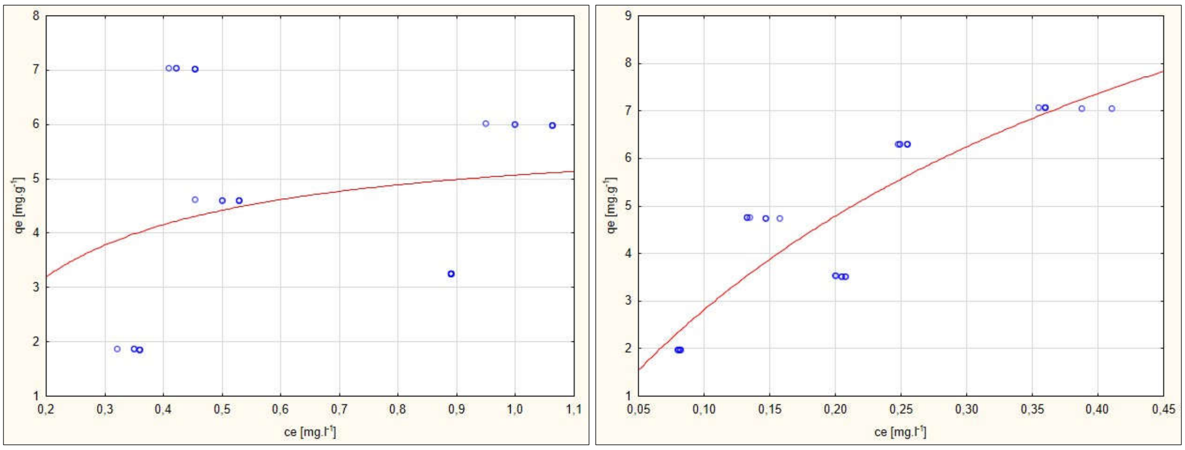

Figure S11. Nonlinear correlations of Cu(II) on bentonite before treatment (left) and after treatment (right) at 10 °C, using the Langmuir isotherm model.

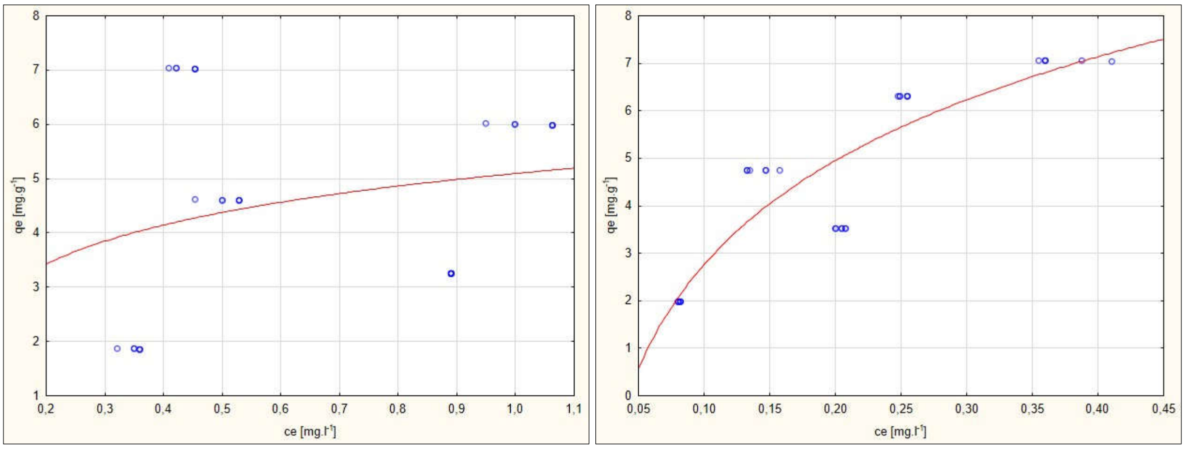

Figure S12. Nonlinear correlations of Cu(II) on bentonite before treatment (left) and after treatment (right) at 10 °C, using the Temkin isotherm model.

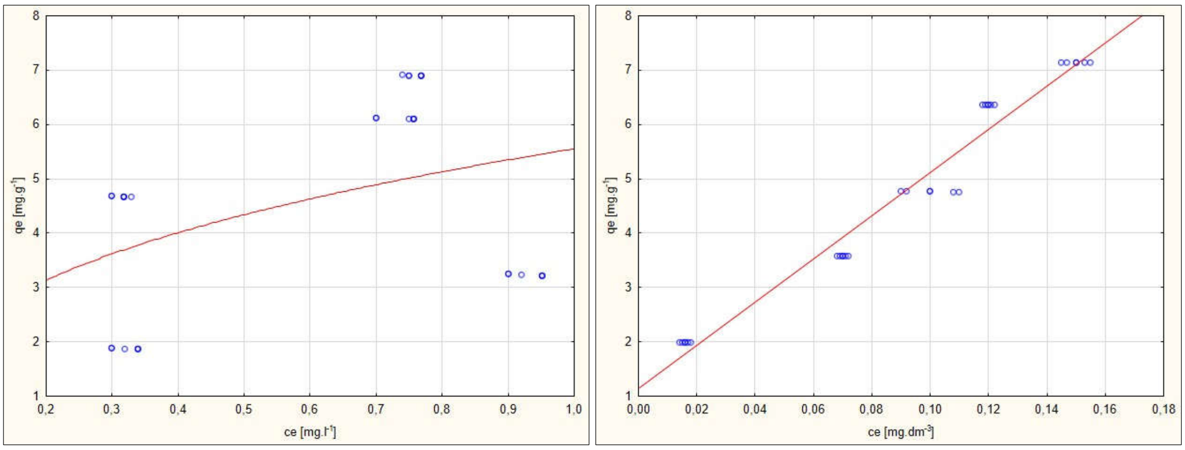

Figure 2. Nonlinear correlations of Cu(II) on bentonite before treatment (left) and after treatment (right) at 20 °C, using the Freundlich isotherm model.

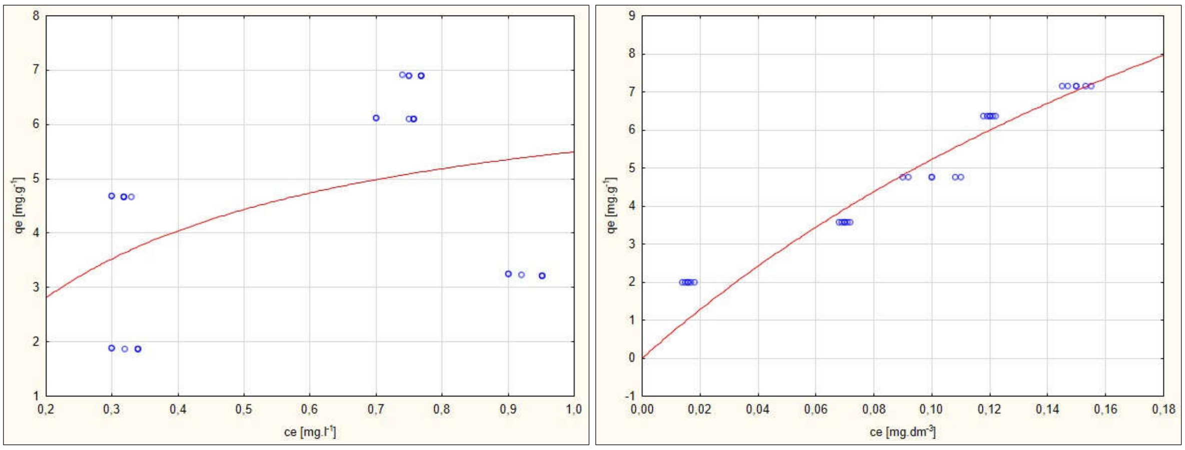

Figure S14. Nonlinear correlations of Cu(II) on bentonite before treatment (left) and after treatment (right) at 20 °C, using the Langmuir isotherm model.

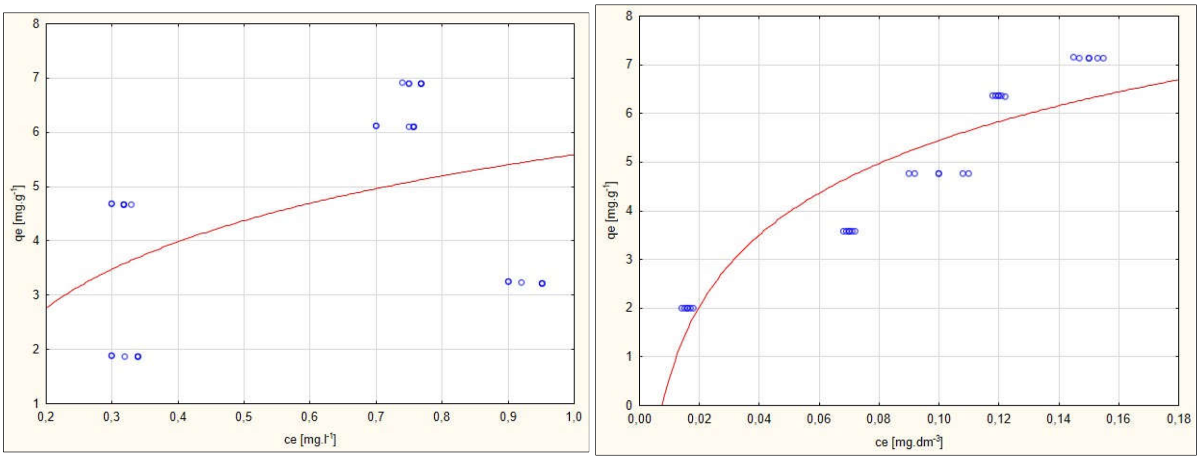

Figure S15. Nonlinear correlations of Cu(II) on bentonite before treatment (left) and after treatment (right) at 20 °C, using the Temkin isotherm model.

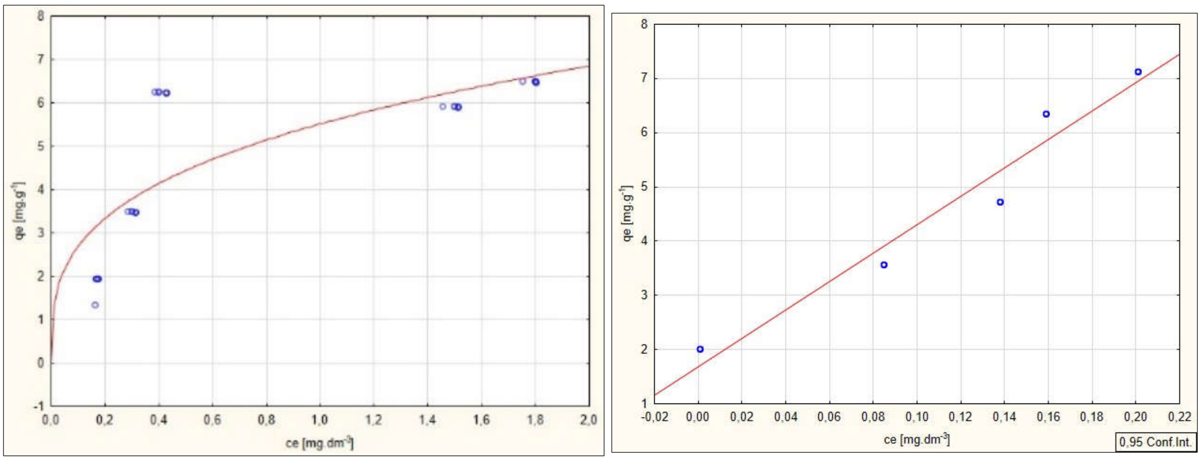

Figure S16. Nonlinear correlations of Cu(II) on bentonite before treatment (left) and after treatment (right) at 30 °C, using the Freundlich isotherm model.

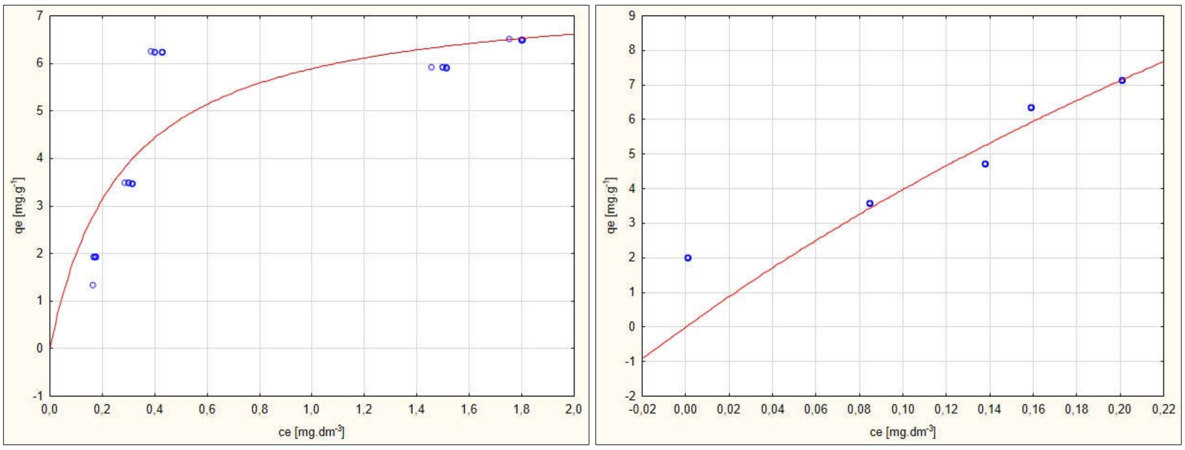

Figure S17. Nonlinear correlations of Cu(II) on bentonite before treatment (left) and after treatment (right) at 30 °C, using the Langmuir isotherm model.

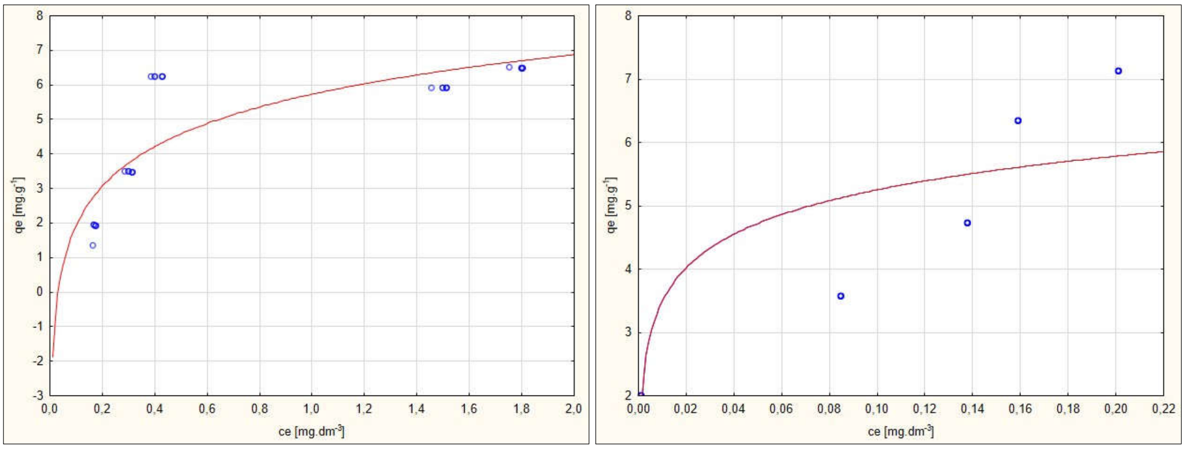

Figure S18. Nonlinear correlations of Cu(II) on bentonite before treatment (left) and after treatment (right) at 30 °C, using the Temkin isotherm model.

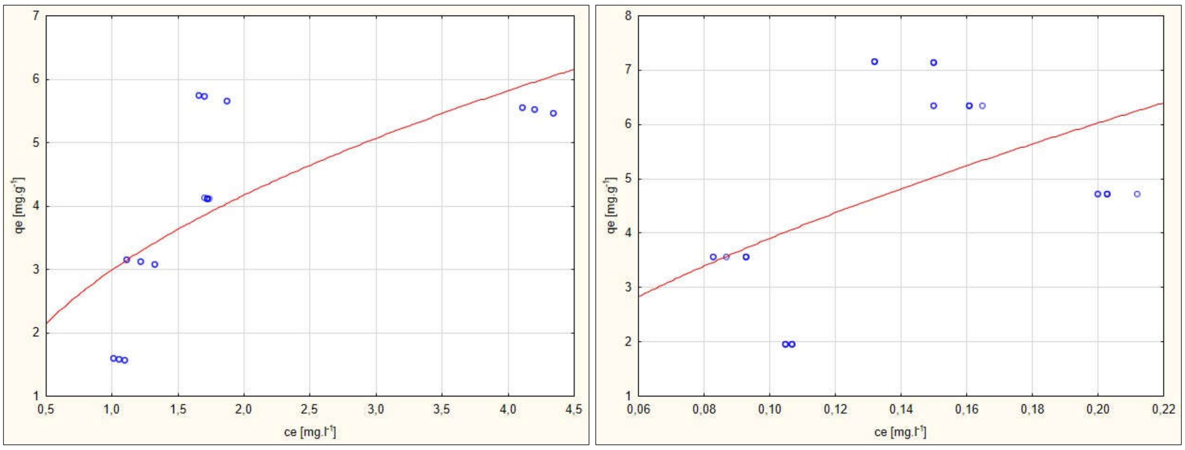

Figure S19. Nonlinear correlations of Cu(II) on zeolite before treatment (left) and after treatment (right) at 10 °C, using the Freundlich isotherm model.

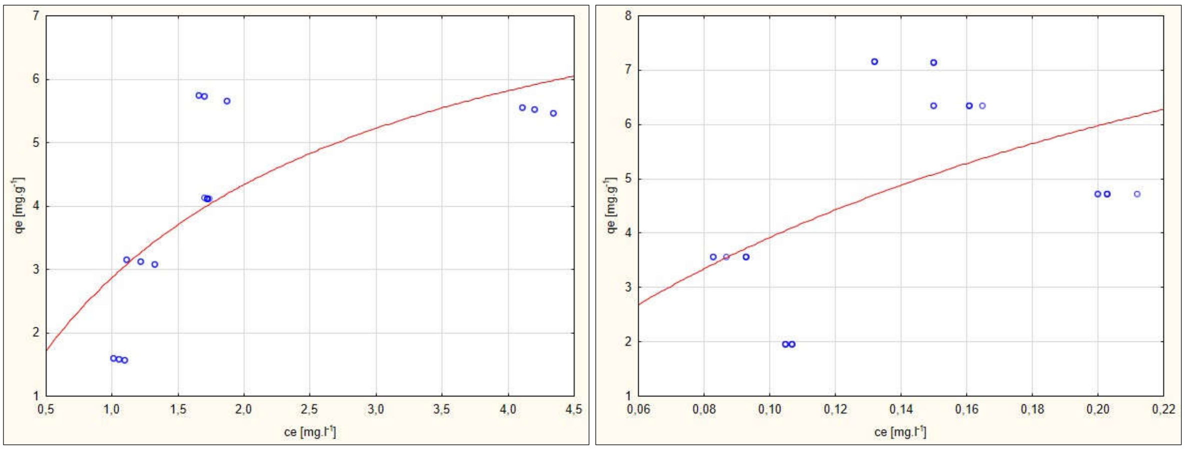

Figure S20. Nonlinear correlations of Cu(II) on zeolite before treatment (left) and after treatment (right) at 10 °C, using the Langmuir isotherm model.

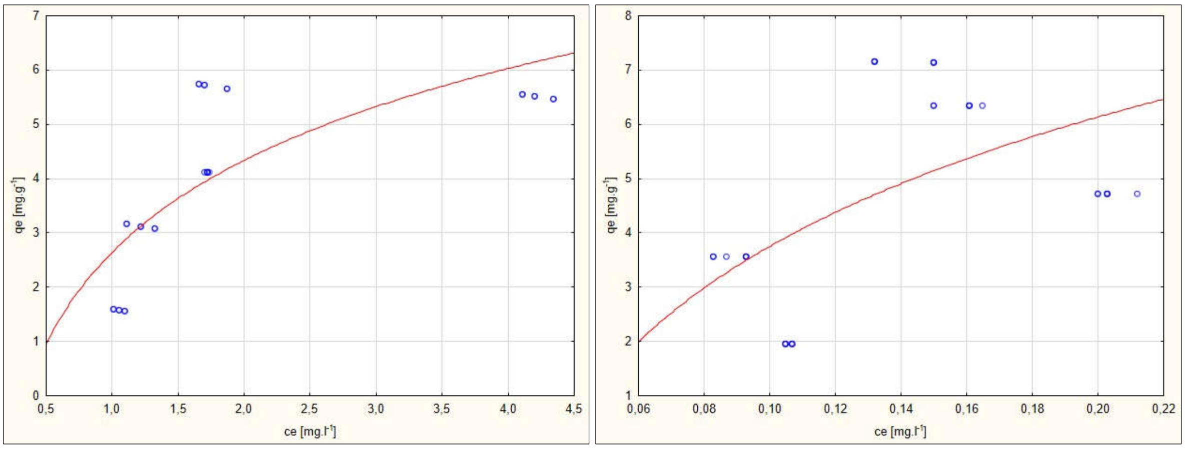

Figure S21. Nonlinear correlations of Cu(II) on zeolite before treatment (left) and after treatment (right) at 10 °C, using the Temkin isotherm model.

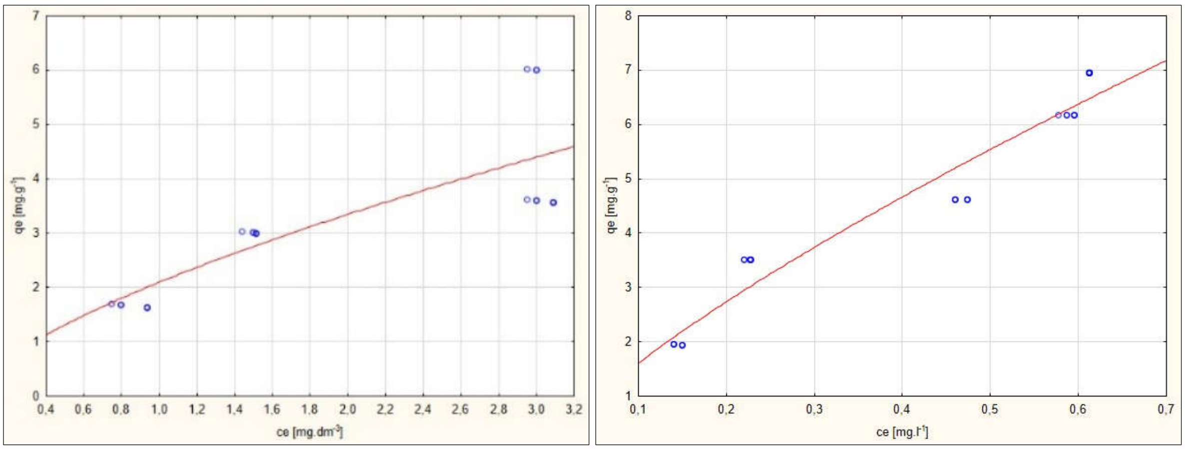

Figure S22. Nonlinear correlations of Cu(II) on zeolite before treatment (left) and after treatment (right) at 20 °C, using the Freundlich isotherm model.

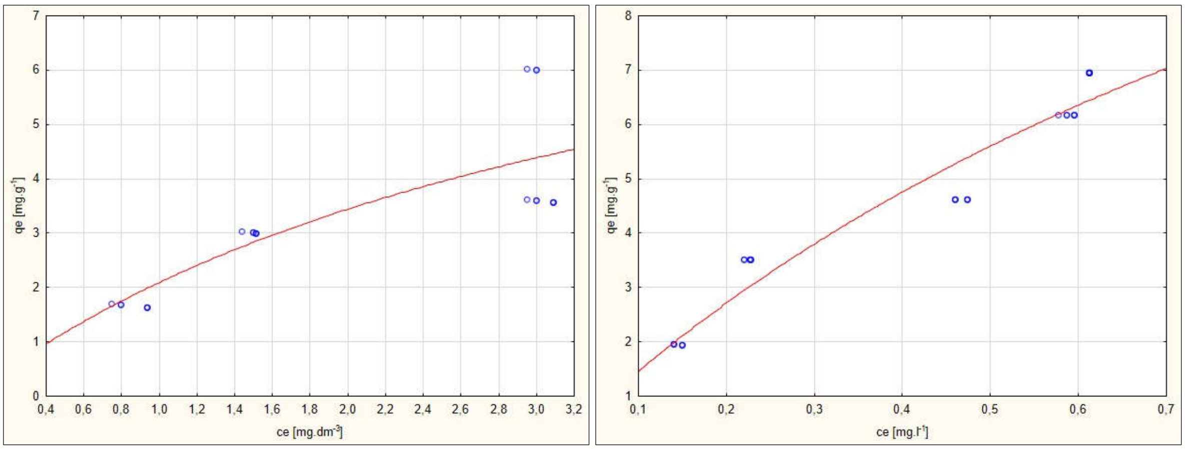

Figure S23. Nonlinear correlations of Cu(II) on zeolite before treatment (left) and after treatment (right) at 20 °C, using the Langmuir isotherm model.

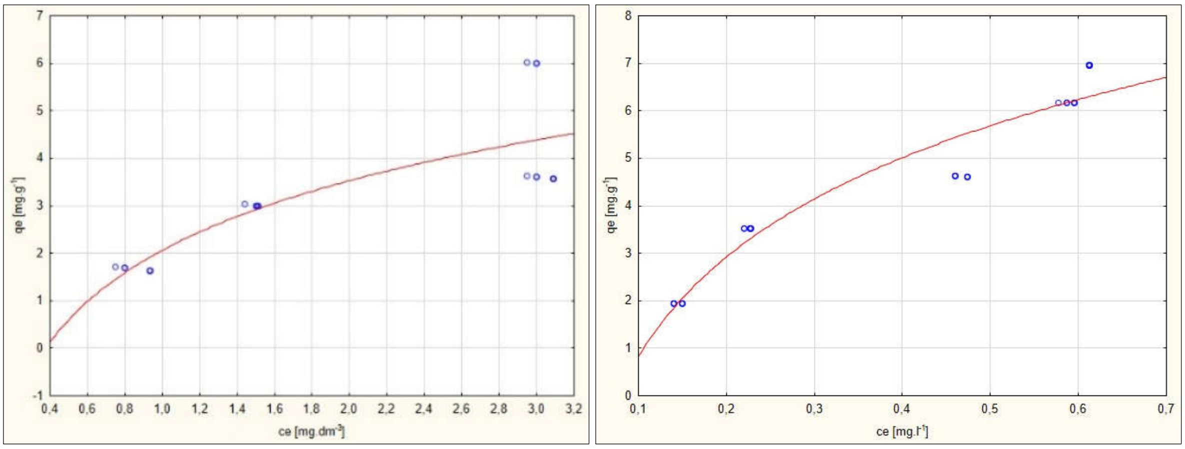

Figure S24. Nonlinear correlations of Cu(II) on zeolite before treatment (left) and after treatment (right) at 20 °C, using the Temkin isotherm model.

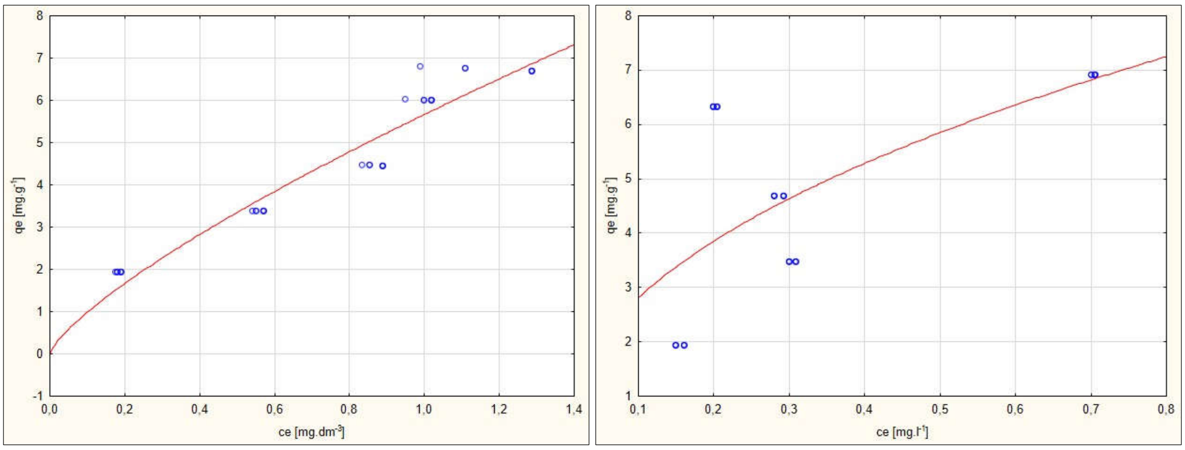

Figure S25. Nonlinear correlations of Cu(II) on zeolite before treatment (left) and after treatment (right) at 30 °C, using the Freundlich isotherm model.

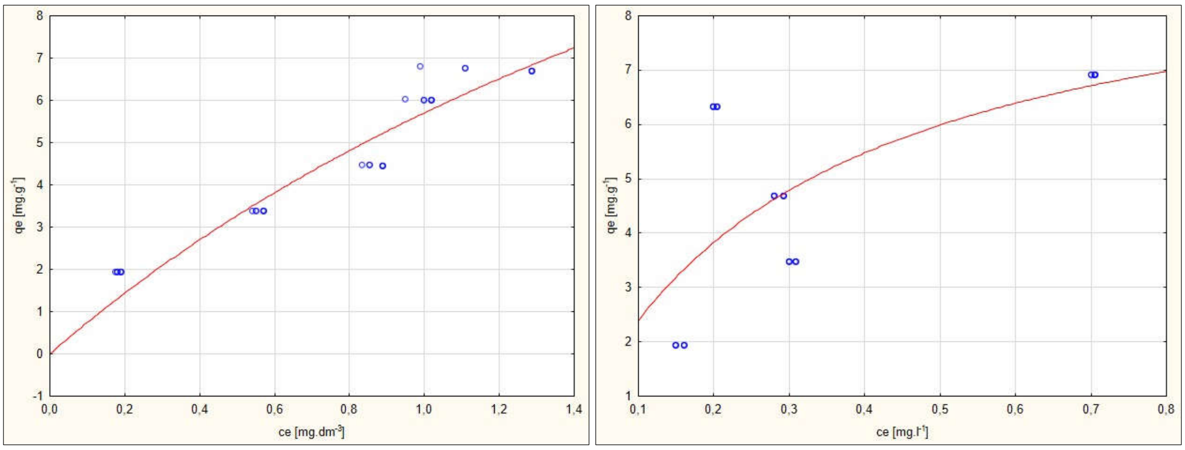

Figure S26. Nonlinear correlations of Cu(II) on zeolite before treatment (left) and after treatment (right) at 30 °C, using the Langmuir isotherm model.

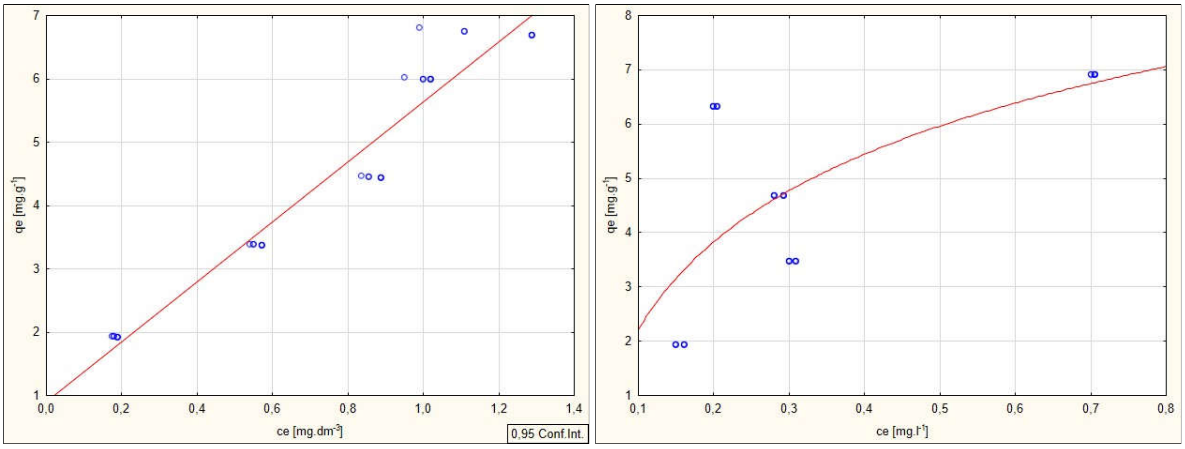

Figure S27. Nonlinear correlations of Cu(II) on zeolite before treatment (left) and after treatment (right) at 30 °C, using the Temkin isotherm model.
